# Metabolic Capabilities of Microorganisms Involved in and Associated with the Anaerobic Oxidation of Methane

**DOI:** 10.3389/fmicb.2016.00046

**Published:** 2016-02-02

**Authors:** Gunter Wegener, Viola Krukenberg, S. Emil Ruff, Matthias Y. Kellermann, Katrin Knittel

**Affiliations:** ^1^Max Planck Institute for Marine MicrobiologyBremen, Germany; ^2^MARUM, Center for Marine Environmental SciencesBremen, Germany; ^3^Department of Earth Science and Marine Science Institute, University of California, Santa BarbaraSanta Barbara, CA, USA

**Keywords:** anaerobic oxidation of methane, archaea, syntrophy, methanogenesis, disproportionation, physiology

## Abstract

In marine sediments the anaerobic oxidation of methane with sulfate as electron acceptor (AOM) is responsible for the removal of a major part of the greenhouse gas methane. AOM is performed by consortia of anaerobic methane-oxidizing archaea (ANME) and their specific partner bacteria. The physiology of these organisms is poorly understood, which is due to their slow growth with doubling times in the order of months and the phylogenetic diversity in natural and *in vitro* AOM enrichments. Here we study sediment-free long-term AOM enrichments that were cultivated from seep sediments sampled off the Italian Island Elba (20°C; hereon called E20) and from hot vents of the Guaymas Basin, Gulf of California, cultivated at 37°C (G37) or at 50°C (G50). These enrichments were dominated by consortia of ANME-2 archaea and Seep-SRB2 partner bacteria (E20) or by ANME-1, forming consortia with Seep-SRB2 bacteria (G37) or with bacteria of the HotSeep-1 cluster (G50). We investigate lipid membrane compositions as possible factors for the different temperature affinities of the different ANME clades and show autotrophy as characteristic feature for both ANME clades and their partner bacteria. Although in the absence of additional substrates methane formation was not observed, methanogenesis from methylated substrates (methanol and methylamine) could be quickly stimulated in the E20 and the G37 enrichment. Responsible for methanogenesis are archaea from the genus *Methanohalophilus* and *Methanococcoides*, which are minor community members during AOM (1–7‰ of archaeal 16S rRNA gene amplicons). In the same two cultures also sulfur disproportionation could be quickly stimulated by addition of zero-valent colloidal sulfur. The isolated partner bacteria are likewise minor community members (1–9‰ of bacterial 16S rRNA gene amplicons), whereas the dominant partner bacteria (Seep-SRB1a, Seep-SRB2, or HotSeep-1) did not grow on elemental sulfur. Our results support a functioning of AOM as syntrophic interaction of obligate methanotrophic archaea that transfer non-molecular reducing equivalents (i.e., via direct interspecies electron transfer) to obligate sulfate-reducing partner bacteria. Additional katabolic processes in these enrichments but also in sulfate methane interfaces are likely performed by minor community members.

## Introduction

In the anoxic marine subsurface large amounts of the potential greenhouse gas methane are formed by microbial and thermal degradation of organic matter. Hence methane is highly abundant in the marine subsurface (Reeburgh, 2007). The efflux of methane from sediments into the water column is however limited, which is mostly due to the effective barrier of methanotrophic microorganisms. The quantitatively most important sink is the coupling of methane oxidation to the reduction of sulfate (AOM) according to the net reaction:

(1) $${\text{CH}}_{4}+{\text{SO}}_{4}^{2-}\to {\text{HCO}}_{3}^{-}+{\text{HS}}^{-}+{\text{H}}_{2}\text{O}$$

with an energy yield of only −34 kJ per mol substrate turnover at standard conditions (Knittel and Boetius, 2009). AOM is performed in dual species microbial consortia of anaerobic methane-oxidizing archaea (ANME), which are closely related to known methanogens, and partner bacteria affiliated to canonical sulfate reducers of the *Desulfosarcina/Desulfococcus* clade (Hinrichs et al., 1999; Boetius et al., 2000; Orphan et al., 2001; Knittel et al., 2005) or of the HotSeep-1 group (Krukenberg et al., under review). Currently three major clades of ANME archaea are known. ANME-2 is the most prominent methanotrophic clade at marine cold gas seeps (Orphan et al., 2002; Mills et al., 2003; Wegener et al., 2008b; Knittel and Boetius, 2009). The temperature at those sites is usually between 4 and 14°C (Knittel and Boetius, 2009). ANME-3 often occurs at mud volcanoes (i.e., Håkon Mosby Mud Volcano; Niemann et al., 2006 *in situ* temperature −1.5°C) and the Eastern Mediterranean seepages (14°C; Omoregie et al., 2008). To our knowledge so far ANME-3 does not proliferate *in vitro*. The third phylogenetic group ANME-1 has been originally described at cold seeps (Hinrichs et al., 1999), but it is particular abundant in diffusive sulfate methane interfaces (Thomsen et al., 2001; Lanoil et al., 2005; Harrison et al., 2009; Aquilina et al., 2010) and in microbial mats and chimney structures at methane seeps in the Black Sea (Michaelis et al., 2002), *in situ* temperature of 10°C. In hydrothermally heated sediments such as in the Guaymas Basin (AOM activity up to 70°C) ANME-1 perform thermophilic methane oxidation (Teske et al., 2002; Holler et al., 2011b; Dowell et al., 2016). All ANME clades form dense consortia with deltaproteobacterial partners, which belong either to Seep-SRB1a from the *Desulfosarcinales/Desulfococcus* subcluster; (Schreiber et al., 2010), Seep-SRB2 from the *Desulfbacterales* subcluster (Kleindienst et al., 2012) or *Desulfobulbus* (mostly ANME-3; Niemann et al., 2006). The partner of thermophilic ANME-1 is HotSeep-1 (Holler et al., 2011b; Wegener et al., 2015). Different naturally enriched AOM communities proliferated *in vitro* (Nauhaus et al., 2002; Krüger et al., 2005; Holler et al., 2009), however cultivation at low temperatures (≤20°C) repeatedly selected for ANME-2, although several source sediments were dominated by other clades (ANME-1 from the Black Sea or ANME-3 at Håkon Mosby Mud Volcano; Holler et al., 2009; own data). The principles underlying this selective growth of ANME-2 *in vitro* have so far not been resolved. Only cultivation at elevated temperatures sustained ANME-1 (Holler et al., 2011b).

The potential of ANME to perform methanogenesis has been repeatedly suggested. This hypothesis based on experiments with natural enrichments (Bertram et al., 2013), on thermodynamic constrains (Alperin and Hoehler, 2009) and on the phylogenetic proximities and genomic similarities of ANME and known methanogens (Lloyd et al., 2011). Furthermore, using radiotracer co-occurrence of AOM and methane formation have been repeatedly measured (Treude et al., 2007; Orcutt et al., 2008) and ANME-1 archaea have been found to be abundant in potentially methanogenic sedimentary horizons (Lloyd et al., 2011). However, tracer transfer from product (DIC) into the reactant pool (methane) might also be explained as inherent process of AOM as suggested by Holler et al. (2011a).

The certainly least understood feature of AOM is how archaea and bacteria interact in the characteristic dual-species consortia. The activation and complete oxidation of methane via a reversal of the well-described methanogenesis pathway can be confidently assigned to the ANME archaea (Hallam et al., 2004; Meyerdierks et al., 2010; Thauer, 2011; Stokke et al., 2012; Wang et al., 2014). The fate of the released electrons including the localization of sulfate reduction is instead so far controversial. Based on their phylogenetic classification as *Deltaproteobacteria* (Knittel et al., 2003; Schreiber et al., 2010; Kleindienst et al., 2012) and the presence of genes and enzymes of sulfate reduction (Milucka et al., 2013; Wegener et al., 2015), all different partner bacteria are likely involved in the sulfur cycle. Different mechanisms for the interaction of ANME and partner bacteria have been suggested. For the sediment-free Isis Mud Volcano AOM enrichment (Mediterranean Sea), incomplete sulfate reduction in ANME-2 and zero-valent sulfur transfer to disproportionating partner bacteria was proposed (Milucka et al., 2012). Instead, for AOM communities in Hydrate Ridge sediments (Coast off Oregon, USA) cytochrome-mediated direct interspecies electron transfer between ANME-2 and their sulfate-reducing partner was proposed (McGlynn et al., 2015). For the interaction of thermophilic ANME-1 and their sulfate-reducing HotSeep-1 partner direct interspecies electron transfer via nanowires and cytochromes was proposed (Wegener et al., 2015).

Here we retrieved three sediment-free AOM enrichments derived from methane-percolated coastal sands off the Mediterranean Island Elba (Italy; enriched at 20°C; E20) as well as a mesophilic enrichment (37°C; G37) and a thermophilic enrichment culture (G50) from the Guaymas Basin. We described community compositions and membrane lipid patterns of these enrichments and performed physiological experiments to test metabolic capabilities attributed to AOM community members including chemoautotrophy, methanogenesis and sulfur disproportionation. Findings were evaluated in further AOM enrichments obtained from different seep sites.

## Material and methods

### Production of AOM enrichments and maintenance

Source material for the E20 enrichment were clastic sediments sampled by scuba diving in 2010 from the coastal hydrocarbon seeps off the Mediterranean island Elba (*in situ* water temperature 12–27°C and 12 m water depth, further described in Ruff et al., this issue). By shaking and collecting the supernatant we concentrated slowly settling microbial biomass from rapidly sinking mineral particles (sand). The concentrated biomass (<1% of the sediment weight) retained 60% of the microbial methane-dependent sulfate reduction rate of the sediment. The G37 and G50 enrichments derived from the methane-rich hydrothermally heated sediments of the Guaymas Basin, Gulf of California, sampled during RV ATLANTIS cruise AT15-56 with the submersible ALVIN in 2009. After determination of applicable cultivation temperatures in a temperature gradient block (Holler et al., 2011b), sediments from distinct temperature horizons have been enriched at the determined temperature optima of AOM (37, 50, 60°C). All enrichments were incubated with marine sulfate reducer medium (Widdel and Bak, 1992) and supplied with methane and sulfate as sole potential redox couple for at least 3 years. Medium was exchanged when sulfide concentrations exceeded 15 mM and biomass was diluted (1:2 or 1:4) when sulfide production exceeded approximately 0.2 mmol l^−1^ day^−1^. Community structures in the 50 and 60°C enrichment were highly similar, thus experiments presented here were performed at 50°C. The additional sample “GF” (here only studied for microbial diversity and sulfur disproportionation), derived from the methane seeps in the vicinity of the Gullfaks oil field in the North Sea and was sampled during RV ALKOR cruise 267 in October 2005 (Wegener et al., 2008b). Sediment-free AOM enrichments from this site were produced by incubation under AOM conditions at room temperature and subsequent dilution as described above. Further methanotrophic enrichment cultures from the Mediterranean (NAUTNIL expedition with RV L Atalante in 2003), from Hydrate Ridge (RV SONNE expedition SO148 in 2000), the Black Sea (RV POSEIDON expedition POS 148 in 2004) and Gulf of Mexico (RV SONNE expedition SO 174), which were only screened for their microbial diversity, were retrieved and cultivated as described (see Supplementary Table 1).

### Cultivation of methanogens from the AOM enrichments

To determine potential methanogenesis activity in the AOM enrichments E20, G37, and G50, triplicate sulfate-free culture aliquots of 10 ml (1:10 dilution) were incubated in 20 ml Hungate tubes with alternative substrates [hydrogen (0.2 MPa), carbon monoxide (0.05 MPa), formate, acetate, methylamine, and methanol (all 20 mM)] for 30 days at their distinct temperature. The development of methane in the headspace was measured using gas chromatography coupled to flame ionization detection (Focus GC, Thermo equipped with a Poropak column; Analytical columns). In this time interval, methane formation was only observed with methylamine and methanol (for both, E20 and G37), but substrates were already fully turned over after 10 days. For these substrates experiments were repeated with more frequent sampling intervals (Figure 4A). Furthermore, for the substrates methanol and methylamine triplicate dilution-to-extinction series with factor 10 dilutions were prepared (down to 10^8^). Methane formation was repeatedly measured and the highest active dilutions (1:10^5^) were further diluted (1:1000). From freeze-thawed pellets of aliquots of these cultures we identified the enriched microbes by direct 16S rRNA gene amplification using the primer pair Arch20F (Massana et al., 1997)/Arc1492R (Teske et al., 2002) and sequencing (PCR and sequencing as described below for AOM enrichments).

### Cultivation of sulfate reducers from the AOM enrichments

To determine potential methane-independent sulfate reduction in the AOM enrichments, triplicate culture aliquots of 10 ml were incubated with possible alternative substrates [hydrogen (0.1 MPa), carbon monoxide (0.05 MPa), methyl sulfide (0.05 MPa) formate, acetate, methylamine, and methanol (all 20 mM)] for 30 days at their distinct temperature. The development of sulfide was measured by a copper sulfate assay and spectroscopic analysis (Cord-Ruwisch, 1985). Dilution-to-extinction series (down to 10^8^ dilution) were set up from active AOM enrichments (G37, G50) with hydrogen as only used alternative electron source, and incubated at their respective temperature for 2 months. After a subsequent second dilution step (1:100) of the highest sulfide-producing dilutions, enriched microbes were identified by 16S rRNA gene amplification (primer pair GM3/GM4; Muyzer et al., 1995) from freeze-thawed pellets of culture aliquots and direct sequencing (PCR and sequencing as described below for AOM enrichments).

### Experiments on alternative sulfur sources in the AOM enrichments

To identify the spectrum of sulfur sources used by the microbial communities in the E20, G37, and G50 AOM enrichments, sulfate-free culture aliquots were incubated with the alternative sulfur sources sulfite (5 mM), thiosulfate (20 mM), and with sulfate (20 mM) as control. Furthermore, we incubated with colloidal (zero-valent) sulfur (ca. 50 mM) that was prepared according to Steudel et al. (1988), and dissolved in anaerobic deionized water (approximately 0.5 mol S^0^ per liter). Triplicate incubations with and without methane (0.2 MPa CH_4_:CO_2_; 90:10) were performed for each substrate. We measured sulfide production calorimetrically using the copper sulfate assay (Cord-Ruwisch, 1985). Dilution-to-extinction series (as described above) were performed for alternative substrates which showed substantial sulfide production (only in zero-valent sulfur enrichment). The highest active dilutions were further diluted (1:100) and enriched microbes were identified by 16S rRNA gene amplification (primer pair GM3/GM4; Muyzer et al., 1995) from freeze-thawed pellets of culture aliquots and direct sequencing (PCR and sequencing conditions as described above for AOM enrichments).

To study the underlying principle of zero-valent sulfur disproportionation, we tested the stoichiometry of sulfur disproportionation by simultaneously measuring sulfate [by ion chromatography; 761 Compact ion chromatograph (Metrohm) with a Metrosep A SUPP 5 column] and sulfide production (copper sulfate assay; Cord-Ruwisch, 1985) in the AOM enrichments and in the zero-valent sulfur enrichments.

### Extraction and analysis of archaeal intact polar lipids from the AOM enrichments

Cell pellets from 30 ml AOM enrichment cultures were spiked with an internal standard (phosphatidylcholine C_21:0_/_21:0_) and 3 g of combusted sand and extracted using a modified Bligh and Dyer protocol (Sturt et al., 2004). The obtained TLEs were stored at −20°C until analyses. IPLs were analyzed by high-performance liquid chromatography electrospray ionization mass spectrometry (HPLC-ESI-MS). Separation of IPLs was achieved on a Dionex Ultimate 3000 UHPLC equipped with a Waters Acquity UPLC BEH amide column (150 × 2.1 mm, 1.8 μm particle size). Chromatographic conditions included constant flow rate of 0.4 ml/min with eluent A [75% acetonitrile; 25% dichloromethane; 0.01% formic acid; 0.01% ammonium hydroxide solution (NH_3_ aq.)] and eluent B [50% methanol 50% Milli-Q water; 0.4% formic acid; 0.4% NH_3_ aq. as previously published (Wörmer et al., 2013]. Under a constant flow, the HPLC routine applied: 99% A and 1% B for 2.5 min, increasing to 5% B at 4 min, followed by a linear gradient to 25% B at 22.5 min and then to 40% B at 26.5 min. Thereafter a 1 min washing step with 40% B followed and afterwards reset to the initial conditions for 8 min to achieve column re-equilibration. Compound detection was conducted on a maXis Ultra-High Resolution qToF-MS (Bruker, Bremen, Germany). IPLs were measured in positive ionization mode scanning a mass-to-charge (m/z) range of 150–2000, with automated data-dependent MS/MS fragmentation of base peak ions. Compound identification was achieved by monitoring exact masses of possible parent ions (present mainly as H^+^ and NH${}_{4}^{+}$ adducts) in combination with characteristic fragmentation patterns (Sturt et al., 2004; Yoshinaga et al., 2011). The reported relative distribution of microbial lipids is based on the peak areas of the respective molecular ions without differentiating for potential differences in response factors; results should therefore be considered as semi-quantitative.

### Determination of microbial carbon sources and growth efficiencies in the AOM enrichments

To determine the role of methane and bicarbonate as carbon sources in the AOM enrichment and their assimilation rate in relation to AOM, we incubated triplicate culture aliquots of E20, G37, and G50 (4 ml) in 5 ml Hungate tubes with ^14^C-bicarbonate [380 kBq, equilibrated with 0.2 MPa CH_4_:CO_2_ or N_2_:CO_2_ (90:10)] or with ^14^C-methane [14 kBq; equilibrated with 0.2 MPa CH_4_:CO_2_ (90:10)]. After 5 days of incubation cell material was transferred to membrane filters (GSWP, 0.2 μm pore size). To remove non-fixed inorganic carbon we washed the filters with saline water (0.5 M NaCl). Potential residual inorganic carbon was removed by exposing the dried filters to a HCl atmosphere for 24 h. Total radioactivity was determined from liquid incubation aliquots (0.1 ml) and incorporated radioactivity was determined from the particulate organic carbon fraction (POC) collected on filters by liquid scintillation counting (scintillation mixture; Filtercount or Permafluor; Perkin Elmer, Waltham, MA, USA; scintillation counter; 2900TR LSA; Packard, Waltham, MA, USA). Counts for ^14^C-compound in POC were corrected for background values. To determine carbon fixation efficiencies (CFE) values were normalized to the added amount of radiotracer and the rate of sulfate reduction

(2) $$\begin{array}{l} \text{CFE}(\%)={[}^{14}\text{C}-{\text{POC}}_{\text{i}}(\text{kBq}){/}^{14}\text{C}-{\text{total}}_{\text{i}}(\text{kBq})\\ \;\times {\text{conc}}_{\text{CS}}(\text{mmol})\times 100]/\text{SRR}(\text{mmol}) \end{array}$$

where ^14^C-POC defines the concentration of radiotracer in the particulate organic carbon (biomass) and ^14^C-total defines the concentrations of added radiotracer (^14^CH_4_ or ^14^C-inorganic carbon) in an experiment “*i*,” and conc_CS_ is the concentration of the carbon source (either methane or inorganic carbon) and the respective sulfate reduction rate (SRR) in replicate vials determined as described below.

### Radiotracer measurement of inorganic carbon fluxes in the AOM enrichments

To track the carbon fluxes between methane and inorganic carbon in AOM enrichments E20, G37, and G50, replicate culture aliquots (4 ml) were incubated in 5 ml Hungate tubes equilibrated with 0.2 MPa CH_4_:CO_2_ (90:10). After 5 days of pre-incubation the vials were completely filled with methane-saturated medium and carrier-free ^14^C-bicarbonate (approximately 66 kBq per sample) or ^35^S-sulfate (100 kBq per sample) was injected into 5 replicates, respectively according to protocols described by Holler et al. (2011a). Concurrently controls inactivated by formaldehyde addition were performed to estimate impurities or abiotic reactions. Samples were incubated for 2 days and reactions were stopped by transferring samples into sodium hydroxide solution (0.5 N) or zinc-acetate solution (20 % w/v), respectively for ^14^C and ^35^S labeling. The ^14^C-bicarbonate and the ^35^SO_4_ samples were processed as described before (Kallmeyer et al., 2004; Holler et al., 2011a). The turnover rates of bicarbonate were inferred by calculating the portion of radiotracer transferred into the methane pool multiplied by the concentration of DIC and divided by the total tracer content in the experiment. Sulfate reduction rates were calculated as described below.

### Sequencing of 16S rRNA gene libraries of AOM enrichments

DNA from AOM enrichments was extracted as described before (Zhou et al., 1996). The protocol encompassed three cycles of freezing and thawing, chemical lysis in a high-salt extraction buffer (1.5 M NaCl) by heating of the suspension in the presence of sodium dodecyl sulfate and hexadecyltrimethyl-ammonium bromide, and treatment with proteinase K, followed by chloroform:isoamylalcohol extraction (24:1) and isopropanol based nucleic acid precipitation. To analyze the phylogeny of the dominant members of the enrichment and to obtain representative full length 16S rRNA gene sequences, the bacterial and archaeal 16S rRNA genes were amplified from the extracted DNA using the primer pair GM3/GM4 (Muyzer et al., 1995) and 20F (Massana et al., 1997)/Arc1492R (Teske et al., 2002), respectively. PCR reaction mixtures were prepared as previously described (Holler et al., 2011b) and subjected to the following cycle conditions: 95°C for 5 min; 26 cycles, each 95°C for 1 min, 46°C (GM3/GM4) or 58°C (Arch20F/Arc1492R) for 1.5 min, and 72°C for 3 min; and a final step at 72°C for 10 min. The amplicons of three replicate PCR reactions were pooled. Following gel electrophoresis bands were extracted from an agarose gel and purified using the QIAquick PCR Purification Kit (Qiagen, Hilden, Germany) according to the manufacturer's recommendations. Purified amplicons were ligated into the pGEM-T Easy vector (Promega, Madison, WI, USA) and transformed into *Escherichia coli* (One Shot Top10 cells; Invitrogen, Carlsbad, CA, USA) following the manufacturer's recommendations. Taq cycle sequencing was performed using ABI BigDye Terminator chemistry and an ABI377 sequencer (Applied Biosystems, Foster City, CA, USA).

### Pyrosequencing of 16S rRNA genes retrieved from AOM enrichments

To obtain an overview about the diversity of rare microbial phyla we performed massive parallel tag sequencing of 10 long-term AOM enrichment cultures. In addition to the four enrichments presented in detail (E20, GB37, GB50, and GF) we also investigated six enrichment cultures that were obtained with samples from Amon Mud Volcano, a Black Sea microbial reef, Black Sea sediments, Caldera Mud Volcano, the Gulf of Mexico, and Hydrate Ridge (see Supplementary Table 1). The latter six enrichment cultures were used for comparison, but are not focus of this study. From DNA that was extracted as described above, we amplified 16S rRNA genes using the primer pairs GM3/907RM (Muyzer et al., 1995, 1998) for bacteria and Arch20F/Arch958RV (Massana et al., 1997; Pires et al., 2012) for archaea. The amplicon libraries were prepared, and the V3–V5 region of these amplicons was sequenced on a 454 Genome Sequencer GS FLX+ (Roche, Basel, Switzerland) at the Max Planck Genome Centre (Cologne, Germany). The raw read data was processed based on a standard operating procedure (Schloss et al., 2011) using Mothur (release 1.33, 02/2014; Schloss et al., 2009). Reads were denoised based on PyroNoise (Quince et al., 2009), trimmed, preclustered (Huse et al., 2010), and chimeras were removed (Edgar et al., 2011). After quality filtering we had a total of 122,363 archaeal and 102,762 bacterial reads forming 13,935 unique archaeal and 17,237 unique bacterial sequences with an average length of 488 and 435 nucleotides, respectively. The alignment and taxonomic classification of the sequences was based on the SILVA small subunit reference database (release 119, 07/2014; Quast et al., 2013). Operational taxonomic units were clustered at 98% 16S rRNA gene V3–V5 sequence identity using average neighbor clustering. The datasets were subsampled to account for unequal sampling effort prior to community analyses and multivariate statistics.

### Comparison of 16S rRNA tags and 16S rRNA gene libraries

To investigate whether the same organisms are present in gene libraries as well as tag datasets we compared the results of the two methods. We made a sequence database of the 16S rRNA gene tags using BLAST (Boratyn et al., 2013) and then searched this database with target 16S rRNA gene sequences from the enrichments. The headers of the resulting output were matched with an OTU_0.02_ list created by mothur (Schloss et al., 2011) to find the sequences that were present in both datasets. We only used sequences that matched the whole length of the sequence and had an *E*-value of basically 0.

### Phylogenetic analysis of retrieved 16S rRNA gene sequences

Retrieved partial 16S rRNA gene sequences (from AOM enrichments and alternative substrate enrichments) were aligned with the SILVA Incremental Aligner (SINA; Pruesse et al., 2007) and classified based on the SILVA small subunit database (release 115; Quast et al., 2013). Phylogenetic analysis was performed with representative, nearly full length (>1200 bp) sequences from AOM enrichments, and from methanogenic, sulfate-reducing and sulfur-disproportionating enrichments (see above) using the ARB software package (Ludwig et al., 2004). Maximum likelihood based trees were calculated by RAxML (Stamatakis, 2006) with GTRCAT as nucleotide substitution model including 235 bacterial and 148 archaeal nearly full length sequences (>1200 bp). A base frequency filter was employed to consider only alignment regions which were at least 50% conserved. 100 bootstrap replicates were used to estimate branch support.

### Nucleotide sequence accession numbers

The 16S rRNA gene sequences were archived in the NCBI public nucleotide sequence databases under the accession numbers KT899714, KT899739-KT899743 (bacteria) and KT899737, KT899738 and KM605124 (archaea). Pyrosequencing raw reads were deposited in the sequence read archive under study accession number SRP065102.

### Catalyzed reporter deposition fluorescence *In situ* hybridization (CARD-FISH)

For CARD-FISH culture aliquots were fixed in 2% formaldehyde for 2 h at room temperature, washed with 1 × phosphate buffered saline (PBS; 8.2 g l^−1^ NaCl, 0.2 g l^−1^, KCl, 1.8 g Na_2_HPO_4_ × 2H_2_O, 0.24 g KH_2_PO_4_, adjusted to pH 7.4) and stored in 1 × PBS:ethanol (1:1) at −20°C. Fixed cells were treated with mild sonication (Sonoplus HD70, Bandelin, Berlin Germany) for 30 s at 10 W and filtered onto GTTP filter (0.2 μm pore size). CARD-FISH was performed as described previously (Pernthaler et al., 2002). For cell wall permeabilization, filters were sequentially incubated in lysozyme solution (10 mg ml^−1^ lysozyme, lyophilized powder (SigmaAldrich) in 0.1 M Tris–HCl, 0.05 M EDTA, pH 8) for 30 min at 37°C, proteinase K solution (15–150 μg l^−1^ proteinase K (Merck) in 0.1 M Tris–HCl, 0.05 M EDTA, 0.5 M NaCl, pH 8) for 2 min at room temperature and optionally in sodium dodecyl sulfate solution (0.5%) for 10 min at room temperature. Endogenous peroxidases were inactivated by incubating the filters in 0.15% H_2_O_2_ in methanol (30 min, room temperature). Oligonucleotide probes were synthesized by Biomers (Ulm, Germany) and applied with formamide concentrations in the hybridization buffer according to literature values. For dual CARD-FISH, peroxidases of the first hybridization were inactivated by 0.3% H_2_O_2_ in methanol (30 min, room temperature). Catalyzed reporter deposition was combined with the fluorochromes Alexa Fluor 488 and Alexa Fluor 594 (Thermo Fisher Scientific). Filters were stained with DAPI (4,6-diamidino-2-phenylindole). Micrographs were obtained by confocal laser scanning microscopy (LSM 780; Zeiss, Oberkochen, Germany).

## Results and discussion

### Cultivation, microbial diversity and archaeal intact polar lipids in the studied enrichment cultures

**Figure 1:**
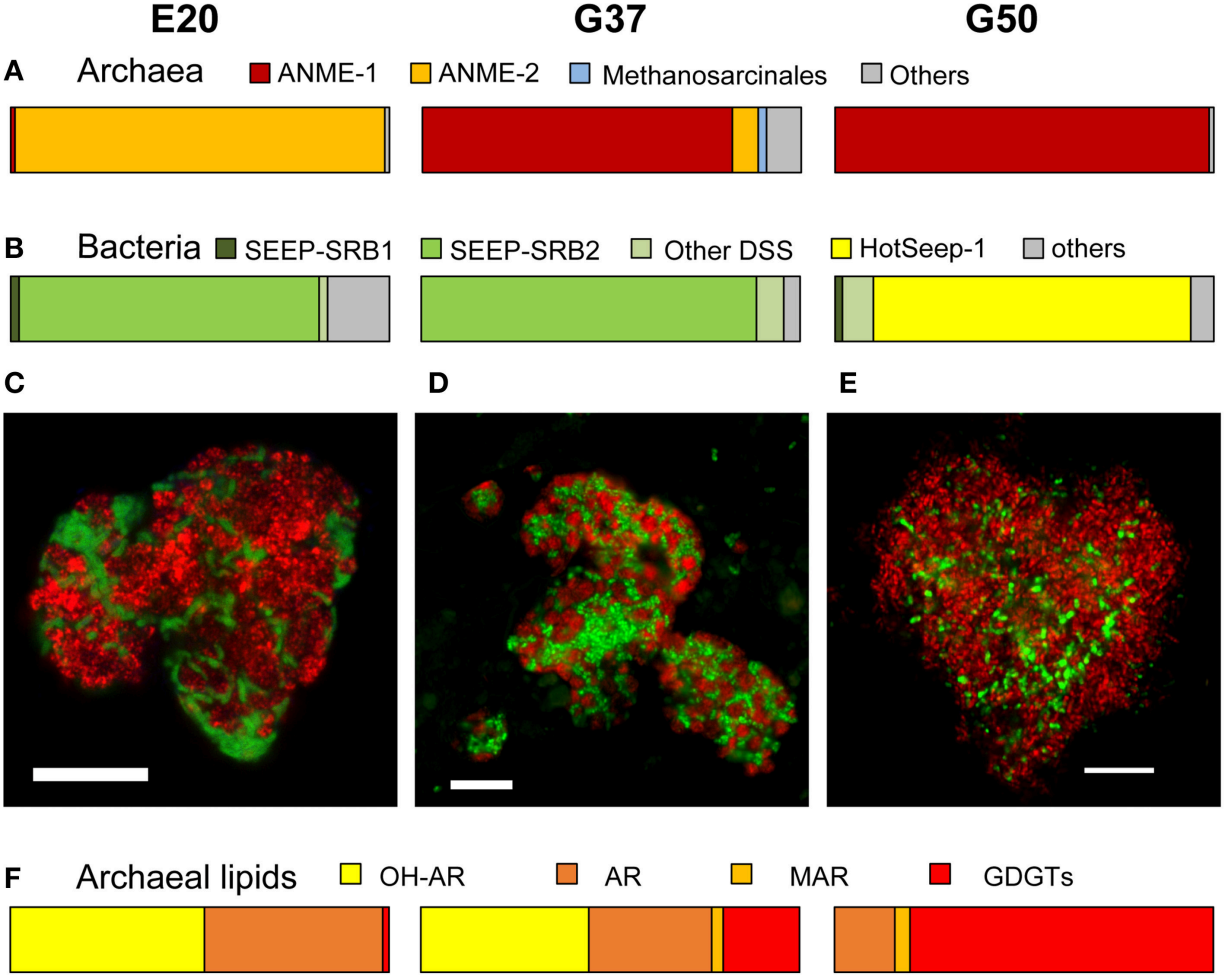
**Comparison of community composition, typical microbial aggregates and archaeal lipids of the three AOM enrichment cultures. (A,B)** Comparison of normalized archaeal and bacterial clone numbers retrieved from the enrichment (for clone number see Table 1; short, badly aligning sequences were not considered here). **(C–E)** Fluorescence *in situ* hybridization of dual-species aggregates in the enrichment (E20: red = ANME-2-538, Treude et al., 2005; green = DSS658, Manz et al., 1998; G37: red = ANME-1-350, Boetius et al., 2000, green = DSS658; G50: red = ANME-1-350, green = HotSeep-1-590, Holler et al., 2011b; bars scale 10 μm). **(F)** Major archaeal membrane intact polar lipid types defined by hydrophobic core groups OH-AR, hydroxyarchaeol; AR, archaeol; MAR, macrocyclic archaeol; GDGT, glyceroldibiphytanylglyceroltetraether. At higher temperatures ANME-1 archaea tend to produce predominantly GDGTs, likely a temperature adaption (for details and 60°C example see Table 2).

The original sediment samples from Guaymas Basin and the Elba seeps showed already high methane-dependent sulfide production when incubated at AOM conditions (about 0.15 μmol g${}_{\text{dw}}^{-1}$; gram dry weight; E20; Ruff et al., this issue to 0.5 and 1.25 μmol g${}_{\text{dw}}^{-1}$ in Guaymas Basin; G37 and G50; Holler et al., 2011b). In E20, cells were separated from the sandy matrix (see Materials and Methods). All samples were further enriched for AOM by cultivation in anoxic marine sulfate-reducer medium equilibrated with 0.225 MPa methane and 0.025 MPa carbon dioxide headspace. Cultivation was performed at the respective temperature optima of 20°C (E20) and 37°C and 50°C (G37/G50). From the development of sulfide production rates and dilution frequencies we estimated doubling times of 69 days (G37) and 55 days (G50; Supplementary Figures 1A,B). Due to repeated subsampling for experiments similar required long-term incubations are yet not available for E20, but we expect doubling times in the range of other cold-adapted enrichments (2–7 month; Girguis et al., 2005; Nauhaus et al., 2005). The studied meso- and thermophilic cultures from Guaymas Basin grew faster than the before studied cold-adapted deep sea AOM enrichments (i.e., 7 month in Hydrate Ridge enrichments; Nauhaus et al., 2007). Hence, after repeated dilution and cultivation a sediment-free state (<100 mg background sediment per liter culture) was reached after 1.5–2 years in the Guaymas Basin cultures. Cultures were maintained at sulfide production rates of 100–250 μmol l${}_{\text{inoculum}}^{-1}$ d^−1^. The microbial composition of the three enrichments were analyzed by sequencing archaeal and bacterial 16S rRNA genes (Figures 1A,B, Table 1, Supplementary Figure 2). In E20 the most sequence-abundant archaeal group was ANME-2 (subgroups ANME-2a, ANME-2b, ANME-2c). Using catalyzed reporter deposition-fluorescence *in situ* hybridization (CARD-FISH) we showed that ANME-2 archaea formed tightly packed consortia with Seep-SRB2 partner bacteria (Figure 1C). G37 consisted of likewise densely packed dual-species consortia of ANME-1 and Seep-SRB2 partner bacteria. (Figure 1D) The dominance of ANME-1 and Seep-SRB2 is typical for moderately heated surface sediments of the Guaymas Basin seeps (Dowell et al., 2016). As shown for 60°C thermophilic AOM enrichments before also the G50 enrichment was dominated by ANME-1 and their partner bacteria HotSeep-1 (Wegener et al., 2015; Figures 1A,B). Compared to the low and medium temperature enrichment cell types were strongly mixed but less densely packed (Figure 1E), which may indicate a less established partnership than in the low-temperature enrichments.

**Table 1:** **Analyzed clones from 16S rRNA gene libraries established from sediment-free methane-oxidizing anaerobic enrichment cultures from Elba and the Guaymas Basin**.

**Phylogenetic group**	**Enrichment**				
	**E20**	**G37**	**G50**	**G60**	**G50 & 60**
**ARCHAEA**					
**Euryarchaeota**					
**Methanomicrobia**					
ANME-1					
ANME-1a		**71 (83%)**	**85 (99%)**	**70 (88%)**	**155 (93%)**
ANME-1b	1 (1%)				
**Methanosarcinales**					
ANME-2					
ANME-2a-2b	**9 (11%)**				
ANME-2b	**23 (30%)**				
ANME-2c	**46 (58%)**	6 (7%)			
Others		3		1	1
Thermoplasmata		6	1	6	7
Thermococci				1	1
Thaumarchaeota				1	1
Crenarchaeota				1	1
Total sequences analyzed	79	86	86	80	166
**BACTERIA**					
**Proteobacteria**					
**Deltaproteobacteria**					
HotSeep-1			**41 (48%)**	**48 (74%)**	**89 (59%)**
Seep-SRB1	7 (9%)		1 (1%)		1 (1%)
Seep-SRB2	**35 (46%)**	**60 (88%)**			
Others	2	5	4		4
Betaproteobacteria				1	1
Bacteroidetes	6				
Spirochaetes	3				
Chloroflexi	1				
Planctomycetes	5				
Firmicutes	3				
Candidate division OP-3			37	3	40
Candidate division OP-8	1			6	6
Candidate division JS1		2			
Others	13	1	3	7	10
Total sequences analyzed	76	68	86	65	151

*Bold numbers represent sequences targeted by probes used for CARD-FISH (Figures 1C–E)*.

Our results of the E20 enrichment and also prior *in vitro* cultivation at low-temperatures (≤20°C; i.e., Hydrate Ridge, Mediterranean seeps such as Amon Mud Volcano, Black Sea; Holler et al., 2009) showed that low-temperature enrichments of mixed communities always led to ANME-2-dominated enrichments (Supplementary Figure 2), whereas ANME-1 is usually not sustained *in vitro*. In contrast, cultivation at elevated temperatures (≥37°C) led to ANME-1-dominated enrichments, even from sites that harbored mixed communities (Table 1, c.f.; Holler et al., 2011b; Kellermann et al., 2012). The different temperature optima and growth ranges of ANME-1 and ANME-2 might be due to their cell membrane structure. The ANME-2 in the E20 enrichments assemble their membranes from double layers of diether lipids (intact archaeols) such as hydroxylated (PG)phosphatidylglycerol archaeol (Figure 1F, Table 2). ANME-1 are instead able to condense diethers to tetraether lipids (Kellermann et al., under review). Hence in the G37 enrichment culture an about 1:1 mixture of diether and tetraether lipids (i.e., glyceroldialkylglyceroltetraether GDGTs) was detected, whereas the high-temperature enrichments (G50 and also G60; the latter only shown in Table 2) contained between 80 and 94% tetraether lipids. The formation of GDGT might allow higher temperature optima (Kellermann et al., 2012) or better resistance in starvation periods (Schouten et al., 2003; Rossel et al., 2008). This observation might also explain the predominance of ANME-1 in most deep sulfate-methane interfaces or in inner parts of microbial chimneys where they have to survive under often minimal substrate concentration. The adaption to harsh conditions or limited substrate availability may, on the other hand, also explain their inability to compete with ANME-2 during cultivation at low temperatures and high substrate availability.

**Table 2:** **Relative composition of archaeal lipids in the three studied enrichments, and for comparison, composition of lipids in G60**.

	**ANME-2**		**ANME-1**	
	**20°C**	**37°C**	**50°C**	**60°C**
**DIETHER LIPIDS**				
**Archaeol-based lipids**				
1Gly-AR	9			
2Gly-AR	18	7	9	3
GN-1G-AR	3			
PG-AR	12	25	6	2
Pent-PG-AR	2			
PE-AR	2			
**Macrocyclic archaeol based lipids**				
PE-MAR		3	4	2
**Hydroxy-archaeol based lipids**				
1Gly-OH-AR	31			
PG-OH-AR	19	1		
PE-OH-AR	1	44		
PI-OH-AR	1			
	98	80	19	7
**TETRAETHER LIPIDS**				
PG-GDGT-PG				
PG-GDGT		5		
1Gly-GDGT	1		1	5
2Gly-GDGT	1	14	79	88
**SUMMARY**				
AR	46	32	15	5
MAR		3	4	2
OH-AR	52	45		
Tetraether	2	20	80	94

*Values given in percent (%). Headgroups: Gly, glycosyl; GN-1G, (N-acetyl)-glucosamine-monoglycosyl; PG, phosphatidylglycerol; PE, phosphatidylethanolamine; PI, phosphatidylinositol; PE, phosphatidylethanolamine. Core lipid: AR, archaeol; MAR, macrocyclic archaeol; OH-AR, hydroxyarchaeol; GDGT, glyceroldibiphytanylglyceroltetraether*.

### Origin of biomass carbon in AOM-performing microbial enrichments

**Figure 2:**
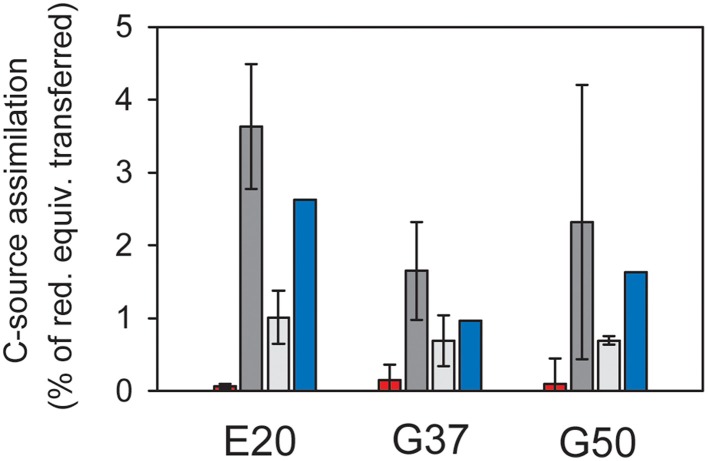
**Assimilation of carbon sources in relation to reducing equivalent transfer assuming an average oxidation state of organic carbon of 0**. Red = methane carbon assimilation; light gray = DIC assimilation in the absence of methane; dark gray = DIC assimilation in the presence of methane; error bars = standard deviation, *n* = 3 per treatment; blue bars = methane-dependent DIC assimilation as difference between incubations with and without methane, therefore no error bars. In all cultures assimilation of inorganic carbon strongly exceeds methane carbon assimilation, suggesting that the latter is likely methane-derived DIC assimilation.

To interpret natural biomass stable isotope signals and to perform stable isotope studies the dominant biomass carbon sources of the active organisms need to be identified. For AOM methane and inorganic carbon have been suggested as carbon sources (Hinrichs et al., 1999; Blumenberg et al., 2005; Wegener et al., 2008a; Kellermann et al., 2012). Here we studied inorganic carbon and methane assimilation into AOM communities using a radiotracer assay with respective labeled carbon sources and tracked the assimilation into the bulk sample. In all three cultures mainly inorganic carbon was assimilated, whereas only 3–15% of the biomass carbon derived from methane (Figure 2). In the absence of methane as energy source, assimilation of inorganic carbon dropped to about 1/10 of the values measured under AOM conditions. This shows that the microbial activity and carbon fixation in the studied cultures strongly depended on the presence of methane and the process of AOM, respectively. During the oxidation of 1 mol methane only 10–40 mM of carbon (mostly of inorganic origin) were incorporated. The rates of inorganic carbon assimilation measured here are in the upper range of growth/ carbon fixation reported in earlier studies (Nauhaus et al., 2007; Wegener et al., 2008a). However, in those studies extremely slow-growing AOM enrichments were investigated with doubling times of approximately 7 months, e.g., for enrichments from Hydrate Ridge.

The predominant use of inorganic carbon as carbon source for assimilation is in line with earlier observations stating “chemoorganoautotrophy” for mesophilic ANME-1 (Kellermann et al., 2012). This growth mode seems to be consistent in cold-adapted and thermophilic methane-oxidizing enrichments. The minor amounts of methane carbon incorporation observed here and in earlier studies (Wegener et al., 2008a) should also be interpreted as assimilation of methane-derived inorganic carbon. The assimilation of methane-derived CO_2_ and further isotope fractionation might also explain the extremely low carbon isotope values. Carbon fixation in ANME proceeds most likely via the acetyl-CoA pathway (Koga and Morii, 2005; Meyerdierks et al., 2010), which causes the highest ^13^C-discrimination (Preuß et al., 1989). It is furthermore consistent with the observation of lowest ^13^C-lipid values in highly active AOM sites, where pore water inorganic carbon derives mostly from methane, thus is also strongly depleted in ^13^C. In less active AOM sites rather moderate ^13^C-signatures of archaeal lipids are observed (Elvert et al., 2005).

### Methanogenesis in the AOM cultures

**Figure 3:**
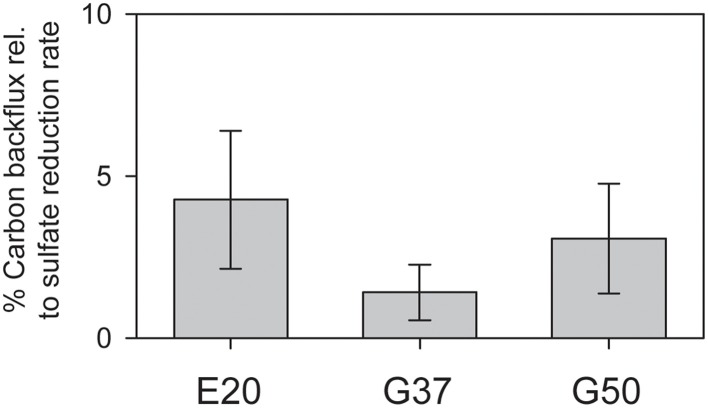
**Production of ^**14**^C-methane from ^**14**^C-bicarbonate relative to AOM rates (here determined by production of ^**35**^S-sulfide from ^**35**^S-sulfate) in the three studied AOM enrichments incubated under AOM conditions at their respective temperature optima (error bars = standard deviation ***n*** = 5 per treatment)**.

Using radiotracer isotope assays (i.e., ^14^CO_2_) transfer of inorganic carbon into the methane pool has been shown for many different AOM systems. This phenomenon has been repeatedly interpreted as capacity of ANME to thrive as methanogens (Orcutt et al., 2008; Lloyd et al., 2011). However, alternatively this tracer transfer was related to enzymatic back reactions. All three studied cultures showed substantial tracer transfer from DIC into the methane pool amounting to 2–5% of the methane-dependent sulfate reduction rate (Figure 3). This tracer transfer is independent of a net formation of methane, as in none of the three cultures methane formation was observed without addition of further methanogenic substrates. Hence, in agreement with earlier hypothesis (Holler et al., 2011a) the observed tracer flux should be seen as intrinsic back reaction during the oxidation of methane in ANME, which proceeds on the same pathways as methanogenesis (Hallam et al., 2004), but is not an energy conserving net reaction.

**Figure 4:**
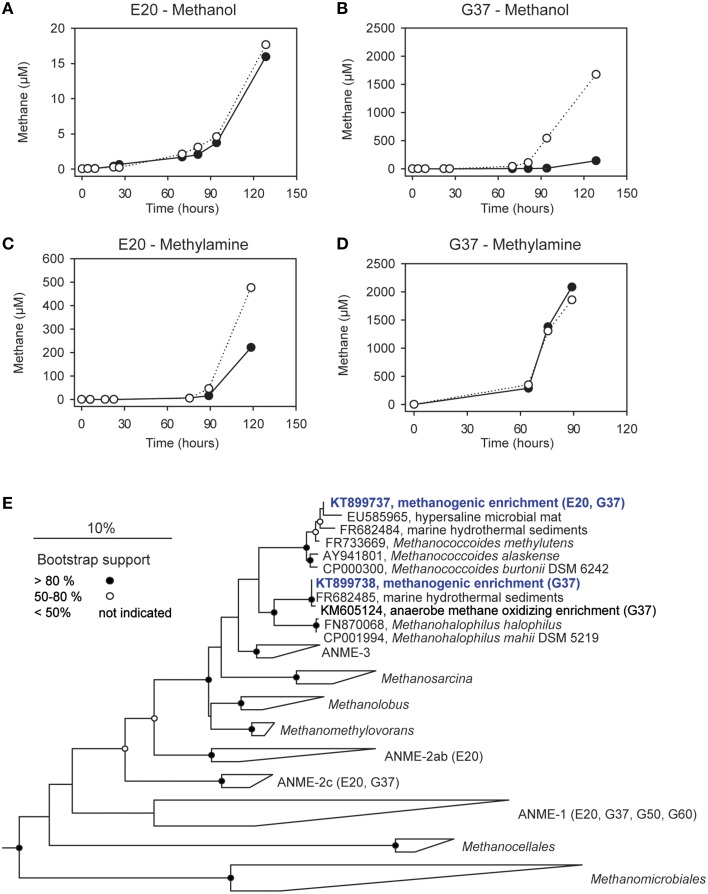
**Methanogenesis and methanogenic archaea in AOM cultures. (A–D)** Methane production in 1:10 dilutions of the E20 and G37 AOM enrichments after addition of methanol or methylamine (10 mM) to the enrichments; open and filled circles, two replicate incubations. **(E)** Phylogenetic affiliation of methanogens (blue) isolated in *dilution-to-extinction* approaches with methanol and methylamine.

We furthermore aimed to induce methanogenesis in the AOM enrichments with typical substrates for methanogens. Therefore, we incubated 1:10 diluted AOM enrichments in sulfate-free medium with different methanogenic substrates and screened those enrichments for methane formation. Hydrogen, acetate and carbon monoxide addition did not cause methane formation in any of the 3 studied enrichments (Table 3), also after extended incubation times of several months (data not shown). However, in the E20 and the G37 cultures methylated substrates (methanol, methylamine) were largely converted to methane within 18 days of incubation (Figures 4A–D). In contrast, the G50 AOM enrichment culture did not show methanogenic activity even after prolonged incubation of 60 days with these two substrates.

**Table 3:** **Stimulation of methanogenesis and sulfate reduction in enrichments from Elba (E20) and Guaymas Basin (G37 and G50) using different substrates (methanogenesis w/o sulfate)**.

**Substrate**	**E20**	**G37**	**G50**
	**Methanogenesis**				
AOM control	+	+	+
No-substrate control	0	0	0
Hydrogen	0	0	0
Formate	0	0	0
Acetate	0	0	0
Methanol	+++	+++	0
Methylamine	+++	+++	0
	**Sulfate reduction**		
AOM control	+	+	+
No-substrate control	0	0	0
Hydrogen	0	++	+++++
Carbon monoxide	0	0	0
Methyl sulfide	0	0	0
Methanol	0	0	0
Acetate	0	0	0
Formate	0	0	0
Propionate	0	0	0

“*+”, expected rate measured; “++”, instant rates low, but rates exceed AOM after longer time; “+++”, instant rate low, but rapidly higher than AOM; “+++++”, rate instantly 3 times higher than during AOM; “0”, no rate detectable*.

Using the *dilution-to-extinction* approach with methylamine or methanol we yielded cultures of methanogenic archaea from the E20 and G37 enrichments. Sequencing of the 16S rRNA gene amplified from the enrichments identified all methylamine cultures as relatives of *Methanococcoides* spp., whereas organisms in methanol cultures were identified as relatives of *Methanohalophilus* spp. (Figure 4E). As methylotrophs, both methanogenic cultures grow on methanol and methylamine. Generally, methylotrophic methanogens grow rapidly, and are hence relatively easy to cultivate (Sowers and Ferry, 1983; Kendall and Boone, 2006). We also retrieved those groups in archaeal 16S rRNA gene tag datasets of the enrichments (Table 4). Both groups contributed between 1 and 3‰ of all archaeal sequences retrieved from the E20 and G37 enrichments. Furthermore, we screened the additional low temperature (4–20°C) methanotrophic enrichment cultures (Supplementary Table 2) for methanogens. All those enrichments contain few but also up to 10‰ sequences that align with *Methanococcoides* or *Methanohalophilus*. In contrast, in the G50 only a single read aligned to *Methanococcoides*. ANME archaea, however, were not enriched in any of the methanogenic enrichments which clearly indicates that ANME cannot thrive as methanogens.

**Table 4:** **Methanogenic and sulfur-disproportionating minor community members**.

OTU0.02^*^	**E20**	**GB 37**	**GB 50**	**GF**	Organism^**^	**Sequence accession number**
A-Otu00017	2.8	1.3	0.1	4.3	*Methanococcoides*	KT899737
A-Otu00024	2.9	1.8	−	7.0	*Methanohalophilus*	KT899738
B-Otu00016	−	−	−	1.0	*Desulfocapsa*	KT899741
B-Otu00114	8.6	−	−	−	Elba-DISP1	KT899742
B-Otu00373	−	2.2	−	−	GB-DISP1	KT899739; KT899740

* Based on 454 pyrosequencing of the 16S rRNA V3-V5 region;

** *presented organisms had a taxonomy quality score of 100; numbers report detected sequences as parts of 1000 (‰)*.

Minor populations of methanogens also regularly appear in sulfate methane interfaces (Wegener et al., 2008b; Ruff et al., 2015), where they likely also thrive on methylated substrates. These substrates (i.e., methanol, methylamines, and methyl sulfides) are not competitively used by other anaerobic microorganisms with potential higher energy yields including sulfate reducers (King, 1984; Kiene et al., 1986; Lovley and Klug, 1986). Yet, the source of methylated substrates in those environments and in the studied laboratory enrichments is unclear. Three possible sources are outlined here: (I) Active ANME may leak methylated compounds, as was shown for aerobic methanotrophs (Xin et al., 2004). This might be in particular true for ANME-1 which lack the methylenetetrahydromethanopterin reductase (Mer) enzyme from a strict reversal of methanogenesis. Meyerdierks and coworkers proposed a bypass via the formation of methanol or methylamine as intermediates which would be oxidized via alcohol dehydrogenases (Meyerdierks et al., 2010). Leakage of these intermediates would be a source of methylated compounds. (II) The strong reversibility of the enzymes involved in AOM, particular of the methyl-CoM reductase (Thauer and Shima, 2008; Holler et al., 2011a) may lead to the formation of trace amounts of methylated substrates. These trace production of methylated substrates should not be confused with the proposal of methyl sulfide as intermediates between ANME and partner bacteria (Moran et al., 2008). Trace production of methylated compounds during AOM or (III) alternatively during decay of microbial biomass might be sufficient to sustain the low numbers of methanogens observed in our enrichments and in sulfate methane interfaces. An experimental detection of these compounds is however challenging as they are efficiently consumed by the methanogenic side communities. In G50 methanogenesis could not be stimulated. The considerably higher maintenance energy at elevated temperatures (Tijhuis et al., 1993) might be the reason for the lack of methanogens and stimulation of methanogenesis here. Our results allow an alternative explanation for the observed stimulation of methane and lipid production in Black Sea mats by methylated compounds as demonstrated by Bertram et al. (2013). This production is unlikely caused by ANME archaea, but should be rather interpreted as growth of specific methanogenic side communities.

### Hydrogenotrophic sulfate reduction and sulfur disproportionation in the AOM enrichments

**Figure 5:**
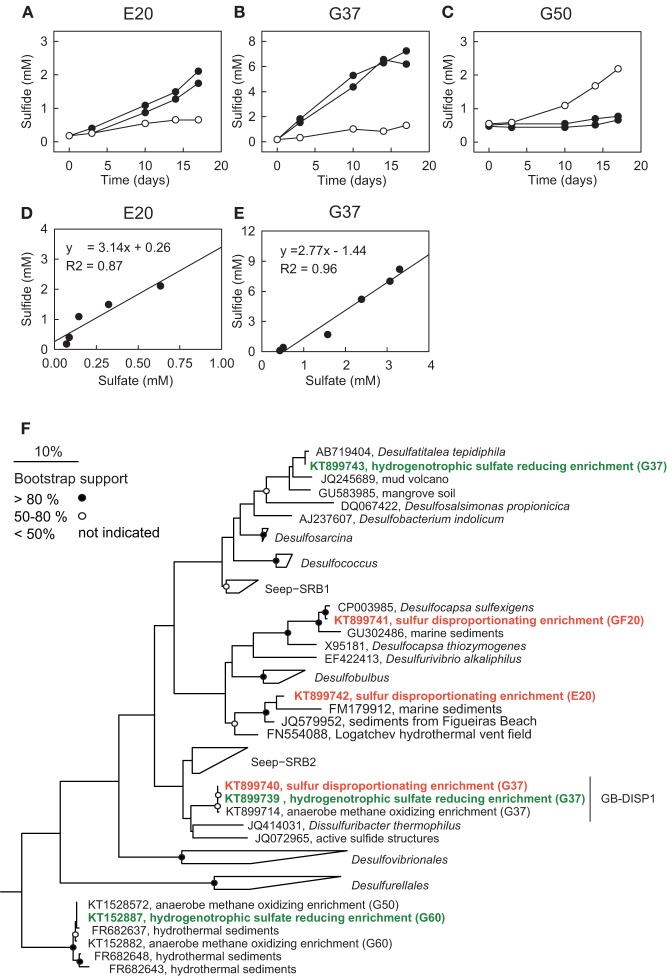
**Sulfur disproportionation and sulfate reduction in AOM enrichment cultures**. **(A–C)** Comparison of developments of sulfide concentrations in the three AOM enrichments under AOM conditions [methane (0.2 MPa) plus sulfate (20 mM; open circles)] and during addition of colloidal sulfur (20 mM; filled circles; two replicates) within 18 days. **(D,E)** Comparison of sulfide and sulfate production in zero-valent sulfur amendments of E20 and G37; disproportionation has not been observed in G50. The observed approximate 3:1 stoichiometry between sulfide and sulfate production is characteristic for disproportionation of elemental (zero-valent) sulfur. **(F)** Phylogenetic affiliation of sulfur-disproportionating (red) and sulfate-reducing (green) bacteria within the Deltaproteobacteria based on nearly full-length 16S rRNA sequences retrieved from high dilutions of AOM-active cultures supplied with elemental sulfur.

We tested the capabilities of the three enrichments to metabolize sulfate with alternative energy sources. As shown before HotSeep-1, the sulfate-reducing bacterium in thermophilic AOM, instantly reacts on hydrogen with elevated sulfide production and growth uncoupled from ANME-1 (Wegener et al., 2015). However, besides G50, also the G37 culture showed sulfide production on hydrogen as substrate. Rates quickly exceeded those of parallel incubations on methane. Following this observation we cultivated the sulfate reducers from the sediment-free AOM enrichment using the *dilution-to-extinction* approach. The retrieved cultures were characterized by direct 16S rRNA gene sequencing. The 16S rRNA gene sequence obtained from one of the cultures affiliated to the larger cluster of Seep-SRB2 bacteria, but was clearly not identical (only 93% sequence similarity which is below the proposed threshold of 94.5% for a genus; Yarza et al., 2014) with the Seep-SRB2 partner bacterium found in this mesophilic and in the cold-adapted AOM culture (Figure 5F). In another hydrogenotrophic culture a bacterium related to *Desulfatitalea tepidiphila* was obtained. The mesophilic *D. tepidiphila* was described to grow as autotroph by hydrogen-dependent sulfate reduction or alternatively by using thiosulfate as electron acceptor and various organic carbon sources as electron donor (Higashioka et al., 2013). Hydrogenotrophic sulfate reduction could not be stimulated in the E20 culture, which likewise is dominated by Seep-SRB2 partner bacteria. Hence it is unlikely that the meso- or psychrophilic AOM partner bacteria can thrive on hydrogen, therewith confirming earlier results which excluded hydrogen as intermediate in low-temperature AOM or as (alternative) substrate of their partner bacteria (Nauhaus et al., 2002).

To investigate the response of the AOM cultures to additions of zero-valent sulfur and therewith to test the observation made by Milucka et al. (2012) in an ANME-2 dominated AOM enrichment derived from Mediterranean mud volcano, we supplied aliquots of the three cultures with freshly prepared colloidal sulfur solution and tracked the development of the chemical endmembers of disproportionation, sulfide and sulfate. As described before (Wegener et al., 2015) sulfur disproportionation was absent in the thermophilic AOM culture (Figure 5C). In contrast, E20 and G37 responded to elemental sulfur addition with rapid sulfide and sulfate production tightly following the 3:1 stoichiometry characteristic for the disproportionation of elemental sulfur (Figures 5D,E). Disproportionation stopped when sulfide concentrations reached approximately 3 mM (E20) or 7 mM (G37). A 7:1 stoichiometry between sulfide and sulfate production, as described for another Mediterranean enrichment (Isis Mud Volcano; cultivated at 20°C and dominated by ANME-2; Milucka et al., 2012) has not been observed in any of our enrichments.

Using a *dilution-to-extinction* approach with colloidal sulfur as only available electron donor we repeatedly isolated specific strains of sulfur-disproportionating bacteria from the two natural AOM enrichments. Interestingly in the dilution series from G37 we repeatedly isolated a single bacterium (hereon called GB-DISP1) that is basically identical to the one isolated on hydrogen (Figure 5F). GB-DISP1 is a rare member in the G37 AOM enrichment, accounting for about 9‰ of the bacterial 16S rRNA gene sequences. The under AOM conditions dominant bacterium Seep-SRB2 however, did not respond to additions of elemental sulfur, hence pointing toward a neutral role of elemental (zero-valent) sulfur in mesophilic AOM proceeding in the G37 enrichment. Growth experiments with the enriched GB-DISP1 showed that it can grow as sulfate-reducing hydrogenotroph (with activity doubling time of 3 days), it can couple sulfur reduction to hydrogen oxidation (activity doubling time 1 day) or it grows as sulfur-disproportionating bacterium (with activity doubling times of about 1 day). Using hydrogen as electron donor GB-DISP1 thrives at sulfide concentrations of up to 20 mM. Instead via sulfur disproportionation GB-DISP1 grows well to sulfide concentrations of up to 5 mM. Above this value sulfide production slows down and sulfide production levels off at around 7 mM. At these sulfide concentrations the energy yield of sulfur disproportionation at 37°C is reduced to approximately −10 kJ mol^−1^ elemental sulfur turnover (Finster, 2008), which is about the minimum free energy yield (ΔG_min_) to sustain microbial metabolism (Hoehler, 2004).

The E20 *dilution-to-extinction* series with elemental sulfur yielded several replicates of a single bacterium, hereon called Elba-DISP1, with high identity to the uncultivated deltaproteobacterial bacterium MSBL7 (Pachiadaki et al., 2014) and the isolated disproportionating species *Desulfurivibrio alkaliphilus* (Sorokin et al., 2008). *D. alkaliphilus* was described as halophilic chemoautotrophic sulfate reducer, capable to thrive on sulfur disproportionation even without supplying a sulfide sink (Poser et al., 2013). Unlike described for *D. alkaliphilus*, we did not succeed to grow Elba-DISP1 as hydrogenotrophic sulfate or elemental sulfur reducer, thus Elba-DISP1 may exclusively thrive as sulfur-disproportionating bacterium. Furthermore, we searched for sulfur disproportionation in the cold seep AOM enrichment culture “GF” retrieved from the Gullfaks oil field (Norwegian North Sea). Indeed also this culture responded to elemental sulfur addition with its disproportionation. The microorganism enriched from the GF culture was *Desulfocapsa sulfoexigens* (>99% 16S rRNA gene identity), one of the first described sulfur-disproportionating microorganism (Finster et al., 1998). In contrast to many other sulfur-disproportionating enrichments we were able to proliferate the enriched sulfur-disproportionating cultures without the addition of iron as sulfide sink as so far only shown for halophiles by Poser et al. (2013). However, due to the limited sulfide tolerance and expected low growth yields of all studied cultures their cell densities remained rather low. We searched for sequences related to disproportionating bacteria in the 16S rRNA gene tag libraries of the other AOM enrichments that proliferated for up to 15 years in the laboratory. The genus *Desulfocapsa* was found in 5 of 10 enrichments, whereas Elba-DISP1 appeared only in the Black Sea enrichment with more than 0.5‰ of the sequences (Supplementary Table 2). Bacteria related to GB-DISP1 were not found in other enrichments than in the GB37 enrichment. Hence we conclude that disproportionating bacteria are a general impurity of AOM enrichment cultures. The abundant partner bacteria, namely Seep-SRB1a, Seep-SRB2 and HotSeep-1, neither respond to elemental sulfur additions nor to any other potential added molecular intermediate (except hydrogen in G50 as discussed above, see Table 3). The absence of stimulation by potential intermediates supports an obligate syntrophic role of Seep-SRB1a, and Seep-SRB2, and an electron transfer in AOM by direct interspecies electron transfer as suggested for thermophilic and psychrophilic AOM (McGlynn et al., 2015; Wegener et al., 2015). Due to the lacking cultivability of these dominant partner bacteria, zero-valent sulfur as primary intermediate exchanged in AOM is less likely.

Sulfur-disproportionating bacteria such as *Desulfocapsa* sp. have also been repeatedly identified in reduced ecosystems and in particular at cold seeps (Lloyd et al., 2006; Sylvan et al., 2012; Ruff et al., 2015). A direct connection of these groups to AOM is meanwhile unlikely in those environments, as they appear in rather low numbers compared to the known partner bacteria (Table 4; Supplementary Table 2). The sulfur source for the disproportionating bacteria in the strictly anaerobic laboratory AOM enrichments is so far unknown. It might be elemental sulfur produced by ANME archaea, which show characteristic sulfur inclusions (Milucka et al., 2012). Furthermore, also the cultivation medium will provide at least trace amounts of elemental sulfur that is produced when sodium sulfide is used as reducing agent. Furthermore, also any leak of oxygen during cultivation will lead to formation of zero-valent sulfur. Activity directly after medium exchange (medium is prepared with about 0.5 mM sulfide) is likely sufficient for the responsible sulfur-disproportionating bacteria to survive later inactivity at increased sulfide concentrations (regular medium change at approximately 12–15 mM sulfide). These short periods of activity are likely sufficient to thrive in the infrequently diluted AOM enrichments. At higher temperatures increased demands of maintenance energy may not have allowed survival of disproportionating bacteria in the G50 culture. In the environment they might thrive on elemental sulfur produced by sulfide-oxidizing bacteria or chemical oxidation of sulfide or being involved in the cryptic sulfur cycle rather below the sulfate methane interfaces (Holmkvist et al., 2011).

## Conclusions

Here we described physiological characteristics of AOM communities at different temperatures from the Elba cold seeps and the Guaymas Basin hydrothermal vent area. We identified inorganic carbon as the dominant carbon source of AOM communities in all three tested AOM cultures, and hence provide additional evidence that all studied ANME and their partner bacteria are autotrophs. Further stable isotope probing experiments should consider this finding with respect to the selection of labeled carbon sources. We found no indications for a capability of ANME to reverse their metabolism towards net methanogenesis. In contrast, we showed the presence of specific known methanogens (*Methanococcoides* spp., *Methanohalophilus* spp.) in all studied low and medium temperature AOM enrichments. Those methanogens can be enriched and isolated using methylated compounds. In the enrichments but also in methane-rich sediments their substrate might be formed as byproduct of AOM or as decay product of AOM biomass. Furthermore, we were able to enrich sulfur-disproportionating bacteria from different non-thermophilic AOM enrichments, which however, are not identical with the known and abundant AOM partner bacteria (Seep-SRB1, Seep-SRB2, or HotSeep-1), but represent known or novel disproportionating bacteria. In the thermophilic (G50) enrichment, neither sulfur disproportionation nor disproportionating taxa were observed. Also the prominent AOM partner bacteria in the studied low and intermediate temperature enrichment did not respond to elemental sulfur addition, which makes transfer of zero-valent sulfur in these enrichments highly unlikely. In summary, we narrowed down metabolic capabilities of the AOM core community, the ANME and their syntrophic partner bacteria. ANME thrive as obligate methane-oxidizing, but autotrophic organisms, which, however, depend on specific partner bacteria that are obligate autotrophic sulfate-reducers. Other metabolic processes observed in AOM cultures and natural enrichments, such as methanogenesis and sulfur disproportionation, are meanwhile likely performed by specialized minor community members.

## Author contributions

GW and VK conceived this study, GW and VK performed microbiological experiments, MK did lipid analysis. GW, VK, SR, and KK performed molecular work and analyzed sequence data. GW and VK wrote the manuscript with input of all authors. All authors read and approved the final version of the manuscript.

## Funding

We would like to thank the Max Planck Society, the German Research Foundation DFG (funding for GW, VK, and SR via Leibniz Grant to Antje Boetius) and the DFG Excellence cluster MARUM (GW) for financial support of this project.

### Conflict of interest statement

The authors declare that the research was conducted in the absence of any commercial or financial relationships that could be construed as a potential conflict of interest.

## References

[B1] Alperin M. J. Hoehler T. M. (2009). Anaerobic methane oxidation by archaea/sulfate-reducing bacteria aggregates: 1. Thermodyn. Phys. Constr. Am. J. Sci. 309, 869–957. 10.2475/10.2009.01

[B2] Aquilina A. Knab N. Knittel K. Kaur G. Geissler A. Kelly S. (2010). Biomarker indicators for anaerobic oxidizers of methane in brackish-marine sediments with diffusive methane fluxes. Org. Geochem. 41, 414–426. 10.1016/j.orggeochem.2009.09.009

[B3] Bertram S. Blumenberg M. Michaelis W. Siegert M. Krüger M. Seifert R. (2013). Methanogenic capabilities of ANME−archaea deduced from 13C−labelling approaches. Environ. Microbiol. 15, 2384–2393. 10.1111/1462-2920.12112 23530864

[B4] Blumenberg M. Seifert R. Nauhaus K. Pape T. Michaelis W. (2005). *In vitro* study of lipid biosynthesis in an anaerobically methane-oxidizing microbial mat. Appl. Environ. Microbiol. 71, 4345–4351. 10.1128/AEM.71.8.4345-4351.2005 16085823 PMC1183335

[B5] Boetius A. Ravenschlag K. Schubert C. J. Rickert D. Widdel F. Gieseke A.. (2000). A marine microbial consortium apparently mediating anaerobic oxidation of methane. Nature 407, 623–626. 10.1038/35036572 11034209

[B6] Boratyn G. M. Camacho C. Cooper P. S. Coulouris G. Fong A. Ma N.. (2013). BLAST: a more efficient report with usability improvements. Nucleic Acids Res. 41, W29–W33. 10.1093/nar/gkt282 23609542 PMC3692093

[B7] Cord-Ruwisch R. (1985). A quick method for the determination of dissolved and precipitated sulfides in cultures of sulfate-reducing bacteria. Microbiol. Meth. 4, 33–36. 10.1016/0167-7012(85)90005-3

[B8] Dowell F. Cardman Z. Dasarathy S. Kellermann M. Lipp J. S. Ruff S. E. (2016). Microbial communities in methane- and short chain alkane-rich hydrothermal sediments of Guaymas Basin. Front. Microbiol. 7:17 10.3389/fmicb.2016.00017 PMC4731509 26858698

[B9] Edgar R. C. Haas B. J. Clemente J. C. Quince C. Knight R. (2011). UCHIME improves sensitivity and speed of chimera detection. Bioinformatics 27, 2194–2200. 10.1093/bioinformatics/btr381 21700674 PMC3150044

[B10] Elvert M. Hopmans E. C. Treude T. Boetius A. Suess E. (2005). Spatial variations of methanotrophic consortia at cold methane seeps: implications from a high-resolution molecular and isotopic approach. Geobiology 3, 195–209. 10.1111/j.1472-4669.2005.00051.x

[B11] Finster K. (2008). Microbiological disproportionation of inorganic sulfur compounds. J. Sulfur Chem. 29, 281–292. 10.1080/17415990802105770

[B12] Finster K. Liesack W. Thamdrup B. (1998). Elemental sulfur and thiosulfate disproportionation by *Desulfocapsa sulfoexigens* sp. nov., a new anaerobic bacterium isolated from marine surface sediment. Appl. Environ. Microbiol. 64, 119–125. 9435068 10.1128/aem.64.1.119-125.1998 PMC124681

[B13] Girguis P. R. Cozen A. E. DeLong E. F. (2005). Growth and population dynamics of anaerobic methane-oxidizing archaea and sulfate-reducing bacteria in a continuous-flow bioreactor. Appl. Environ. Microbiol. 71, 3725–3733. 10.1128/AEM.71.7.3725-3733.2005 16000782 PMC1169053

[B14] Hallam S. J. Putnam N. Preston C. M. Detter J. C. Rokhsar D. Richardson P. M.. (2004). Reverse methanogenesis: testing the hypothesis with environmental genomics. Science 305, 1457–1462. 10.1126/science.1100025 15353801

[B15] Harrison B. K. Zhang H. Berelson W. Orphan V. J. (2009). Variations in archaeal and bacterial diversity associated with the sulfate-methane transition zone in continental margin sediments (Santa Barbara Basin, California). Appl. Environ. Microbiol. 75, 1487–1499. 10.1128/AEM.01812-08 19139232 PMC2655439

[B16] Higashioka Y. Kojima H. Watanabe M. Fukui M. (2013). *Desulfatitalea tepidiphila* gen. nov., sp. nov., a sulfate-reducing bacterium isolated from tidal flat sediment. Int. J. Syst. Evol. Microbiol. 63, 761–765. 10.1099/ijs.0.043356-0 22581901

[B17] Hinrichs K. U. Hayes J. M. Sylva S. P. Brewer P. G. DeLong E. F. (1999). Methane-consuming archaebacteria in marine sediments. Nature 398, 802–805. 10.1038/19751 10235261

[B18] Hoehler T. M. (2004). Biological energy requirements as quantitative boundary conditions for life in the subsurface. Geobiology 2, 205–215. 10.1111/j.1472-4677.2004.00033.x

[B19] Holler T. Wegener G. Knittel K. Boetius A. Brunner B. Kuypers M. M. M. (2009). Substantial 13C/12C and D/H fractionation during anaerobic oxidation of methane by marine consortia enriched *in vitro*. Environ. Microbiol. Rep. 1, 370–376. 10.1111/j.1758-2229.2009.00074.x 23765889

[B20] Holler T. Wegener G. Niemann H. Deusner C. Ferdelman T. G. Boetius A.. (2011a). Carbon and sulfur back flux during anaerobic microbial oxidation of methane and coupled sulfate reduction. Proc. Natl. Acad. Sci. U.S.A. 108, E1484–E1490. 10.1073/pnas.1106032108 22160711 PMC3248532

[B21] Holler T. Widdel F. Knittel K. Amann R. Kellermann M. Y. Hinrichs K. U.. (2011b). Thermophilic anaerobic oxidation of methane by marine microbial consortia. ISME J. 5, 1946–1956. 10.1038/ismej.2011.77 21697963 PMC3223311

[B22] Holmkvist L. Ferdelman T. G. Jørgensen B. B. (2011). A cryptic sulfur cycle driven by iron in the methane zone of marine sediment (Aarhus Bay, Denmark). Geochim. Cosmoch. Acta 75, 3581–3599. 10.1016/j.gca.2011.03.033

[B23] Huse S. M. Welch D. M. Morrison H. G. Sogin M. L. (2010). Ironing out the wrinkles in the rare biosphere through improved OTU clustering. Environ. Microbiol. 12, 1889–1898. 10.1111/j.1462-2920.2010.02193.x 20236171 PMC2909393

[B24] Kallmeyer J. Ferdelman T. G. Weber A. Fossing H. Jorgensen B. B. (2004). A cold chromium distillation procedure for radiolabeled sulfide applied to sulfate reduction measurements. Limnol. Oceanogr. Methods 2, 171–180. 10.4319/lom.2004.2.171

[B25] Kellermann M. Y. Wegener G. Elvert M. Yoshinaga M. Y. Lin Y. S. Holler T.. (2012). Autotrophy as a predominant mode of carbon fixation in anaerobic methane-oxidizing microbial communities. Proc. Natl. Acad. Sci. U.S.A. 109, 19321–19326. 10.1073/pnas.1208795109 23129626 PMC3511159

[B26] Kendall M. M. Boone D. R. (2006). The Order Methanosarcinales. The Prokaryotes. New York, NY: Springer, 244–256

[B27] Kiene R. P. Oremland R. S. Catena A. Miller L. G. Capone D. G. (1986). Metabolism of reduced methylated sulfur compounds in anaerobic sediments and by a pure culture of an estuarine methanogen. Appl. Environ. Microbiol. 52, 1037–1045. 16347202 10.1128/aem.52.5.1037-1045.1986 PMC239170

[B28] King G. M. (1984). Utilization of hydrogen, acetate, and “noncompetitive”; substrates by methanogenic bacteria in marine sediments. Geomicrobiol. J. 3, 275–306. 10.1080/01490458409377807

[B29] Kleindienst S. Ramette A. Amann R. Knittel K. (2012). Distribution and *in situ* abundance of sulfate-reducing bacteria in diverse marine hydrocarbon seep sediments. Environ. Microbiol. 14, 2689–2710. 10.1111/j.1462-2920.2012.02832.x 22882476

[B30] Knittel K. Boetius A. (2009). Anaerobic oxidation of methane: progress with an unknown process. Annu. Rev. Microbiol. 63, 311–334. 10.1146/annurev.micro.61.080706.093130 19575572

[B31] Knittel K. Lösekann T. Boetius A. Kort R. Amann R. (2005). Diversity and distribution of methanotrophic archaea at cold seeps. Appl. Environ. Microbiol. 71, 467–479. 10.1128/AEM.71.1.467-479.2005 15640223 PMC544223

[B32] Knittel K. Boetius A. Lemke A. Eilers H. Lochte K. Pfannkuche O. (2003). Activity, distribution, and diversity of sulfate reducers and other bacteria in sediments above gas hydrate (Cascadia margin, Oregon). Geomicrobiol. J. 20, 269–294. 10.1080/01490450303896

[B33] Koga Y. Morii H. (2005). Recent advances in structural research on ether lipids from archaea including comparative and physiological aspects. Biosci. Biotech. Bioch. 69, 2019–2034. 10.1271/bbb.69.2019 16306681

[B34] Krüger M. Treude T. Wolters H. Nauhaus K. Boetius A. (2005). Microbial methane turnover in different marine habitats. Palaeogeogr. Palaeocl. 227, 6–17. 10.1016/j.palaeo.2005.04.031

[B35] Lanoil B. D. La Duc M. T. Wright M. Kastner M. Nealson K. H. Bartlett D. (2005). Archaeal diversity in ODP legacy borehole 892b and associated seawater and sediments of the Cascadia Margin. FEMS Microbiol. Ecol. 54, 167–177. 10.1016/j.femsec.2005.03.015 16332316

[B36] Lloyd K. G. Lapham L. Teske A. (2006). An anaerobic methane-oxidizing community of ANME-1b archaea in hypersaline Gulf of Mexico sediments. Appl. Environ. Microbiol. 72, 7218–7230. 10.1128/AEM.00886-06 16980428 PMC1636178

[B37] Lloyd K. G. Alperin M. J. Teske A. (2011). Environmental evidence for net methane production and oxidation in putative ANaerobic MEthanotrophic (ANME) archaea. Environ. Microbiol. 13, 2548–2564. 10.1111/j.1462-2920.2011.02526.x 21806748

[B38] Lovley D. R. Klug M. J. (1986). Model for the distribution of sulfate reduction and methanogenesis in freshwater sediments. Geochim. Cosmochim. Acta 50, 11–18. 10.1016/0016-7037(86)90043-8

[B39] Ludwig W. Strunk O. Westram R. Richter L. Meier H. Yadhukumar. (2004). ARB: a software environment for sequence data. Nucleic Acids Res. 32, 1363–1371. 10.1093/nar/gkh293 14985472 PMC390282

[B40] Manz W. Eisenbrecher M. Neu T. R. Szewzyk U. (1998). Abundance and spatial organization of Gram-negative sulfate-reducing bacteria in activated sludge investigated by *in situ* probing with specific 16S rRNA targeted oligonucleotides. FEMS Microbiol. Ecol. 25, 43–61. 10.1111/j.1574-6941.1998.tb00459.x

[B41] Massana R. Murray A. E. Preston C. M. DeLong E. F. (1997). Vertical distribution and phylogenetic characterization of marine planktonic Archaea in the Santa Barbara Channel. Appl. Environ. Microbiol. 63, 50–56. 8979338 10.1128/aem.63.1.50-56.1997 PMC168301

[B42] McGlynn S. E. Chadwick G. L. Kempes C. P. Orphan V. J. (2015). Single cell activity reveals direct electron transfer in methanotrophic consortia. Nature 526, 531–535. 10.1038/nature15512 26375009

[B43] Meyerdierks A. Kube M. Kostadinov I. Teeling H. Glöckner F. O. Reinhardt R.. (2010). Metagenome and mRNA expression analyses of anaerobic methanotrophic archaea of the ANME-1 group. Environ. Microbiol. 12, 422–439. 10.1111/j.1462-2920.2009.02083.x 19878267

[B44] Michaelis W. Seifert R. Nauhaus K. Treude T. Thiel V. Blumenberg M.. (2002). Microbial reefs in the Black Sea fueled by anaerobic oxidation of methane. Science 297, 1013–1015. 10.1126/science.1072502 12169733

[B45] Mills H. J. Hodges C. Wilson K. MacDonald I. R. Sobecky P. A. (2003). Microbial diversity in sediments associated with surface-breaching gas hydrate mounds in the Gulf of Mexico. FEMS Microbiol. Ecol. 46, 39–52. 10.1016/S0168-6496(03)00191-0 19719581

[B46] Milucka J. Widdel F. Shima S. (2013). Immunological detection of enzymes for sulfate reduction in anaerobic methane-oxidizing consortia. Environ. Microbiol. 15, 1561–1571. 10.1111/1462-2920.12003 23095164

[B47] Milucka J. Ferdelman T. G. Polerecky L. Franzke D. Wegener G. Schmid M.. (2012). Zero-valent sulphur is a key intermediate in marine methane oxidation. Nature 491, 541–546. 10.1038/nature11656 23135396

[B48] Moran J. J. Beal E. J. Vrentas J. M. Orphan V. J. Freeman K. H. House C. H. (2008). Methyl sulfides as intermediates in the anaerobic oxidation of methane. Environ. Microbiol. 10, 162–173. 10.1111/j.1462-2920.2007.01441.x 17903217

[B49] Muyzer G. Teske A. Wirsen C. O. Jannasch H. W. (1995). Phylogenetic relationships of *Thiomicrospira* species and their identification in deep-sea hydrothermal vent samples by denaturing gradient gel electrophoresis of 16S rDNA fragments. Arch. Microbiol. 164, 165–172. 10.1007/BF02529967 7545384

[B50] Muyzer G. Brinkhoff T. Nübel U. Santegoeds C. Schäfer H. Wawer C. (1998). Denaturing gradient gel electrophoresis (DGGE) in microbial ecology, in Mol Microb Ecol Man, eds Kowalchuk G. A. de Bruijn F. J. I. M. H. Akkermans A. D. van Elsas J. D. (New York, NY: Springer), 2645–2671.

[B51] Nauhaus K. Boetius A. Krüger M. Widdel F. (2002). *In vitro* demonstration of anaerobic oxidation of methane coupled to sulphate reduction in sediments from a marine gas hydrate area. Environ. Microbiol. 4, 296–305. 10.1046/j.1462-2920.2002.00299.x 12080959

[B52] Nauhaus K. Treude T. Boetius A. Krüger M. (2005). Environmental regulation of the anaerobic oxidation of methane: a comparison of ANME-I and ANME-II communities. Environ. Microbiol. 7, 98–106. 10.1111/j.1462-2920.2004.00669.x 15643940

[B53] Nauhaus K. Albrecht M. Elvert M. Boetius A. Widdel F. (2007). *In vitro* cell growth of marine archaeal-bacterial consortia during anaerobic oxidation of methane with sulfate. Environ. Microbiol. 9, 187–196. 10.1111/j.1462-2920.2006.01127.x 17227423

[B54] Niemann H. Lösekann T. de Beer D. Elvert M. Nadalig T. Knittel K. (2006). Novel microbial communities of the Haakon Mosby mud volcano and their role as a methane sink. Nature 443, 854–858. 10.1038/nature05227 17051217

[B55] Omoregie E. O. Mastalerz V. de Lange G. Straub K. L. Kappler A. Røy H.. (2008). Biogeochemistry and community composition of iron- and sulfur-precipitating microbial mats at the Chefren mud volcano (Nile Deep Sea Fan, Eastern Mediterranean). Appl. Environ. Microbiol. 74, 3198–3215. 10.1128/AEM.01751-07 18378658 PMC2394935

[B56] Orcutt B. Samarkin V. Boetius A. Joye S. (2008). On the relationship between methane production and oxidation by anaerobic methanotrophic communities from cold seeps of the Gulf of Mexico. Environ. Microbiol. 10, 1108–1117. 10.1111/j.1462-2920.2007.01526.x 18218032

[B57] Orphan V. J. House C. H. Hinrichs K. U. McKeegan K. D. DeLong E. F. (2001). Methane-consuming archaea revealed by directly coupled isotopic and phylogenetic analysis. Science 293, 484–487. 10.1126/science.1061338 11463914

[B58] Orphan V. J. House C. H. Hinrichs K. U. McKeegan K. D. DeLong E. F. (2002). Multiple archaeal groups mediate methane oxidation in anoxic cold seep sediments. Proc. Natl. Acad. Sci. U.S.A. 99, 7663–7668. 10.1073/pnas.072210299 12032340 PMC124316

[B59] Pachiadaki M. G. Yakimov M. M. LaCono V. Leadbetter E. Edgcomb V. (2014). Unveiling microbial activities along the halocline of Thetis, a deep-sea hypersaline anoxic basin. ISME J. 8, 2478–2489. 10.1038/ismej.2014.100 24950109 PMC4260694

[B60] Pernthaler A. Pernthaler J. Amann R. (2002). Fluorescence *in situ* hybridization and catalyzed reporter deposition for the identification of marine bacteria. Appl. Environ. Microbiol. 68, 3094–3101. 10.1128/AEM.68.6.3094-3101.2002 12039771 PMC123953

[B61] Pires A. C. Cleary D. F. Almeida A. Cunha A. Dealtry S. Mendonca-Hagler L. C.. (2012). Denaturing gradient gel electrophoresis and barcoded pyrosequencing reveal unprecedented archaeal diversity in mangrove sediment and rhizosphere samples. Appl. Environ. Microbiol. 78, 5520–5528. 10.1128/AEM.00386-12 22660713 PMC3406151

[B62] Poser A. Lohmayer R. Vogt C. Knoeller K. Planer-Friedrich B. Sorokin D.. (2013). Disproportionation of elemental sulfur by haloalkaliphilic bacteria from soda lakes. Extremophiles 17, 1003–1012. 10.1007/s00792-013-0582-0 24030483

[B63] Preuß A. Schauder R. Fuchs G. Stichler W. (1989). Carbon isotope fractionation by autotrophic bacteria with three different CO_2_ fixation pathways. Z Naturforsch B 44, 397–402. 10.1515/znc-1989-5-610

[B64] Pruesse E. Quast C. Knittel K. Fuchs B. M. Ludwig W. Peplies J.. (2007). SILVA: a comprehensive online resource for quality checked and aligned ribosomal RNA sequence data compatible with ARB. Nucleic Acids Res. 35, 7188–7196. 10.1093/nar/gkm864 17947321 PMC2175337

[B65] Quast C. Pruesse E. Yilmaz P. Gerken J. Schweer T. Yarza P.. (2013). The SILVA ribosomal RNA gene database project: improved data processing and web-based tools. Nucleic Acids Res. 41, D590–D596. 10.1093/nar/gks1219 23193283 PMC3531112

[B66] Quince C. Lanzén A. Curtis T. P. Davenport R. J. Hall N. Head I. M.. (2009). Accurate determination of microbial diversity from 454 pyrosequencing data. Nat. Methods 6, 639–641. 10.1038/nmeth.1361 19668203

[B67] Reeburgh W. (2007). Oceanic methane biogeochemistry. Chem. Rev. 107, 486–513. 10.1021/cr050362v 17261072

[B68] Rossel P. E. Lipp J. S. Fredricks H. F. Arnds J. Boetius A. Elvert M. (2008). Intact polar lipids of anaerobic methanotrophic archaea and associated bacteria. Org. Geochem. 39, 992–999. 10.1016/j.orggeochem.2008.02.021

[B69] Ruff S. E. Biddle J. F. Teske A. P. Knittel K. Boetius A. Ramette A. (2015). Global dispersion and local diversification of the methane seep microbiome. Proc. Natl. Acad. Sci. U.S.A. 112, 4015–4020. 10.1073/pnas.1421865112 25775520 PMC4386351

[B70] Schloss P. D. Gevers D. Westcott S. L. (2011). Reducing the effects of PCR amplification and sequencing artifacts on 16S rRNA-based studies. PLoS ONE 6:e27310. 10.1371/journal.pone.0027310 22194782 PMC3237409

[B71] Schloss P. D. (2009). Introducing mothur: open-source, platform-independent, community-supported software for describing and comparing microbial communities. Appl. Environ. Microbiol. 75, 7537–7541. 10.1128/AEM.01541-09 19801464 PMC2786419

[B72] Schouten S. Wakeham S. G. Hopmans E. C. Damsté J. S. S. (2003). Biogeochemical evidence that thermophilic archaea mediate the anaerobic oxidation of methane. Appl. Environ. Microbiol. 69, 1680–1686. 10.1128/AEM.69.3.1680-1686.2003 12620859 PMC150050

[B73] Schreiber L. Holler T. Knittel K. Meyerdierks A. Amann R. (2010). Identification of the dominant sulfate-reducing bacterial partner of anaerobic methanotrophs of the ANME-2 clade. Environ. Microbiol. 12, 2327–2340. 10.1111/j.1462-2920.2010.02275.x 21966923

[B74] Sorokin D. Y. Tourova T. P. Mussmann M. Muyzer G. (2008). *Dethiobacter alkaliphilus* gen. nov. sp. nov., and *Desulfurivibrio alkaliphilus* gen. nov. sp. nov.: two novel representatives of reductive sulfur cycle from soda lakes. Extremophiles 12, 431–439. 10.1007/s00792-008-0148-8 18317684

[B75] Sowers K. R. Ferry J. G. (1983). Isolation and characterization of a methylotrophic marine methanogen, *Methanococcoides methylutens* gen. nov., sp. nov. Appl. Environ. Microbiol. 45, 684–690. 16346215 10.1128/aem.45.2.684-690.1983 PMC242344

[B76] Stamatakis A. (2006). RAxML-VI-HPC: maximum likelihood-based phylogenetic analyses with thousands of taxa and mixed models. Bioinformatics 22, 2688–2690. 10.1093/bioinformatics/btl446 16928733

[B77] Steudel R. Göbel T. Holdt G. (1988). The molecular composition of hydrophilic sulfur sols prepared by decomposition of thiosulfate. Z Naturforsch B 43, 203–218. 10.1515/znb-1988-0212

[B78] Stokke R. Roalkvam I. Lanzen A. Haflidason H. Steen I. H. (2012). Integrated metagenomic and metaproteomic analyses of an ANME-1-dominated community in marine cold seep sediments. Environ. Microbiol. 14, 1333–1346. 10.1111/j.1462-2920.2012.02716.x 22404914

[B79] Sturt H. F. Summons R. E. Smith K. Elvert M. Hinrichs K. U. (2004). Intact polar membrane lipids in prokaryotes and sediments deciphered by high-performance liquid chromatography/electrospray ionization multistage mass spectrometry—new biomarkers for biogeochemistry and microbial ecology. Rapid Commun. Mass Sp 18, 617–628. 10.1002/rcm.1378 15052572

[B80] Sylvan J. B. Toner B. M. Edwards K. J. (2012). Life and death of deep-sea vents: bacterial diversity and ecosystem succession on inactive hydrothermal sulfides. MBio 3, e00279–e00211. 10.1128/mBio.00279-11 22275502 PMC3262234

[B81] Teske A. Hinrichs K. U. Edgcomb V. de Vera Gomez A. Kysela D. Sylva S. P.. (2002). Microbial diversity of hydrothermal sediments in the Guaymas Basin: evidence for anaerobic methanotrophic communities. Appl. Environ. Microbiol. 68, 1994–2007. 10.1128/AEM.68.4.1994-2007.2002 11916723 PMC123873

[B82] Thauer R. K. (2011). Anaerobic oxidation of methane with sulfate: on the reversibility of the reactions that are catalyzed by enzymes also involved in methanogenesis from CO_2_. Curr. Opin. Microbiol. 14, 292–299. 10.1016/j.mib.2011.03.003 21489863

[B83] Thauer R. K. Shima S. (2008). Methane as fuel for anaerobic microorganisms. Ann. N.Y. Acad. Sci. 1125, 158–170. 10.1196/annals.1419.000 18096853

[B84] Thomsen T. R. Finster K. Ramsing N. B. (2001). Biogeochemical and molecular signatures of anaerobic methane oxidation in a marine sediment. Appl. Environ. Microbiol. 67, 1646–1656. 10.1128/AEM.67.4.1646-1656.2001 11282617 PMC92781

[B85] Tijhuis L. Van Loosdrecht M. C. Heijnen J. J. (1993). A thermodynamically based correlation for maintenance gibbs energy requirements in aerobic and anaerobic chemotrophic growth. Biotechnol. Bioeng. 42, 509–519. 10.1002/bit.260420415 18613056

[B86] Treude T. Knittel K. Blumenberg M. Seifert R. Boetius A. (2005). Subsurface microbial methanotrophic mats in the Black Sea. Appl. Environ. Microbiol. 71, 6375–6378. 10.1128/AEM.71.10.6375-6378.2005 16204560 PMC1265934

[B87] Treude T. Orphan V. Knittel K. Gieseke A. House C. H. Boetius A. (2007). Consumption of methane and CO2 by methanotrophic microbial mats from gas seeps of the anoxic Black Sea. Appl. Environ. Microbiol. 73, 2271–2283. 10.1128/AEM.02685-06 17277205 PMC1855681

[B88] Wang F.-P. Zhang Y. Chen Y. He Y. Qi J. Hinrichs K.-U.. (2014). Methanotrophic archaea possessing diverging methane-oxidizing and electron-transporting pathways. ISME J. 8, 1069–1078. 10.1038/ismej.2013.212 24335827 PMC3996691

[B89] Wegener G. Niemann H. Elvert M. Hinrichs K. U. Boetius A. (2008a). Assimilation of methane and inorganic carbon by microbial communities mediating the anaerobic oxidation of methane. Environ. Microbiol. 10, 2287–2298. 10.1111/j.1462-2920.2008.01653.x 18498367

[B90] Wegener G. Krukenberg V. Riedel D. Tegetmeyer H. E. Boetius A. (2015). Intercellular wiring enables electron transfer between methanotrophic archaea and bacteria. Nature 526, 587–590. 10.1038/nature15733 26490622

[B91] Wegener G. Shovitri M. Knittel K. Niemann H. Hovland M. Boetius A. (2008b). Biogeochemical processes and microbial diversity of the Gullfaks and Tommeliten methane seeps (Northern North Sea). Biogeosciences 5, 1127–1144. 10.5194/bg-5-1127-2008

[B92] Widdel F. Bak F. (1992). Gram-negative mesophilic sulfate-reducing bacteria, in The Prokaryotes, Vol. 4, eds Balows A. T. H. Dworkin M. Harder W. Schleifer K. H. (Berlin; Heidelberg; New York, NY: Springer), 3352–3378.

[B93] Wörmer L. Lipp J. S. Schröder J. M. Hinrichs K. U. (2013). Application of two new LC–ESI–MS methods for improved detection of intact polar lipids (IPLs) in environmental samples. Org. Geochem. 59, 10–21. 10.1016/j.orggeochem.2013.03.004

[B94] Xin J.-Y, Cui, J.-R, Niu, J.-Z, Hua, S.-F, Xia, C.-G, Li, S.-M. (2004). Production of methanol from methane by methanotrophic bacteria. Biocatal. Biotransfor. 22, 225–229. 10.1080/10242420412331283305

[B95] Yarza P. Yilmaz P. Pruesse E. Glöckner F. O. Ludwig W. Schleifer K.-H.. (2014). Uniting the classification of cultured and uncultured bacteria and archaea using 16S rRNA gene sequences. Nat. Rev. Microbiol. 12, 635–645. 10.1038/nrmicro3330 25118885

[B96] Yoshinaga M. Y. Kellermann M. Y. Rossel P. E. Schubotz F. Lipp J. S. Hinrichs K.-U. (2011). Systematic fragmentation patterns of archaeal intact polar lipids by high-performance liquid chromatography/electrospray ionization ion-trap mass spectrometry. Rapid Commun. Mass Sp 25, 3563–3574. 10.1002/rcm.5251 22095505

[B97] Zhou J. Bruns M. A. Tiedje J. M. (1996). DNA recovery from soils of diverse composition. Appl. Environ. Microbiol. 62, 316–322. 8593035 10.1128/aem.62.2.316-322.1996 PMC167800

