# Supplementary material for: Metabolic Capabilities of Microorganisms Involved in and Associated with the Anaerobic Oxidation of Methane
Source: Front Microbiol. 2016 Feb 2;7:46. doi: 10.3389/fmicb.2016.00046 (PMC4736303; doi:10.3389/fmicb.2016.00046)
Supplement: Supplementary file 1 [file DataSheet1.pdf]

Supplementary Material

Metabolic capabilities of microorganisms involved in and associated with the anaerobic oxidation of methane

Gunter Wegener*, Viola Krukenberg, S. Emil Ruff, Matthias Y. Kellermann, Katrin Knittel

*** Correspondence:** Gunter Wegener: gwegener@mpi-bremen.de

1. Supplementary Data description

Here we provide an overview on the different AOM enrichment cultures including their origin, the respective acknowledged scientific expeditions and applied basic cultivation conditions (Supplementary Table 1). Supplementary Fig. 1 provides an overview on the development of methane-dependent sulfide production rates and the resulting activity doubling times of the two Guaymas Basin AOM enrichment cultures. Supplementary Fig. 2 displays the phylogenetic affiliation and relative sequence abundance of archaeal and bacterial clades identified in all 10 studied AOM enrichment cultures. Supplementarty Table 2 shows the relative sequence abundance of specific known methanotrophic (ANME) and methanogenic archaea, and of known AOM partner bacteria and of sulfur-disproportinating bacteria identified in the sediment-free long-term AOM enrichments.

Our results show that long-term, sediment-free AOM enrichments are dominted by typical AOM organisms (ANME clades and their partner bacteria). However all enrichments still harbor a large diversity of different archaea (i.e. *Thermoplasmatales* or MBGD) and bacteria (i.e. *Anaerolinaceae, Cladithrix* or relatives of the Candidate divisions JS1, OD1 and OP11). The function of these minor communty members is so far unknown, however they are most likely heterotrophs feeding on exudates of AOM. Nearly all studied cultures contain sequences of methylotrophic methanogens and disproportionating bacteria. These organisms are rare during AOM conditions (<<1%). However, when cultures are supplying with specific substrates for methanogens (i.e. methanol, methylamine) or disproportionationg bacteria (i.e. colloidal sulfur), these groups can be rapidly enriched.

2. Supplementary Tables and Figures

Supplementary Table 1. Origin, sampling depth and sampling year of different AOM enrichment cultures. *Temperature (Temp) describes the *in vitro* incubation temperature.

Sample	Acro-nym	Location	water depth	Site	Samp-ling	Temp. (°C)*	Sampling campaign
Guaymas50	G50	27.01 N 111.41 W	1999m	Hydrothermal vent	2009	50	RV Atlantis exp. AT15-45 with submarine Alvin, Chief Scientist A. Teske
Guaymas37	G37	27.01 N 111.41 W	1999m	Hydrothermal vent	2009	37	
Elba Seep	E20	42.74 N 010.12 E	12m	Cold Seep	2010	20	Hydra Diving Station, PI Miriam Weber
Gullfaks Seep	GF	61.17 N 002.24 E	150m	Cold Seep	2005	20	RV Alkor Cruise 267; Chief Scientist A. Boetius
Amon MV	AMV	31.73 N 032.37 E	1250m	Mud Volcano	2003	20	NAUTINIL exp. RV L Atalante, CS J.-P. Foucher
Black Sea mat	BSM	44.74 N 031.97 E	300m	Microbial Chimney	2004	12	RV POSEIDON exp. 317/3 with submersible JAGO, Chief Scientist. B.B. Joergensen (METROL)
Black Sea culture	BSC	44.74 N 031.97 E	300m	Microbial Chimney	2004	4	
Menes Caldera	MC	32.11 N 028.16 E	3018m	Cold Seep	2003	20	NAUTINIL exp. RV L Atalante, Jean-Paul Foucher
Hydrate Ridge	HR	44.55 N 125.25 W	780m	Cold Seep	2000	12	RV SONNE Exp. SO-148; CS E.Suess, P. Linke
Gulf of Mexico	GoM	27.74 N 091.31 W	504m	Cold Seep	2003	20	RV Sonne exp. 174, CS: G. Bohrmann OTEGA

Supplementary Table 2: Relative sequence abundance of methantrophic and methanogenic archaeal clades, known AOM partner bacteria and disproportionating bacteria in 10 studied AOM enrichment cultures. Values normalized to parts of 1000 (‰) of obtained archaeal and bacterial 16S rRNA gene sequences, respectively.

Organism	E20	G37	G50	GF	AMV	BSC	BSM	MC	GoM	HR
Archaea										
ANME2a/2b	217	8	10	616	390	177	517	878	528	76
ANME-2c	457	6	7	11	276	601	152	23	87	343
ANME-1	7	15*	805	7	6	5	7	7	5	5
ANME-1a	2	12	87	1	2	2	2	2	2	2
Methanococcoides	3	2	0	5	5	5	2	10	6	2
Methanohalophilus	3	2	0	8	1	0	1	2	3	0
Bacteria										
SEEP-SRB1a	9	6	58	435	56	205	109	245	158	161
SEEP-SRB2	83*	383	3	7	25	4	1	4	30	35
HotSeep-1	1	2	270	2	1	1	2	2	2	3
Desulfocapsa	0	0	0	1	22	0	0	1	69	6
Elba-DISP1	10	0	0	0	0	1	0	0	0	0
GB-DISP1	0	2	0	0	0	0	0	0	0	0

* likely strongly underestimated abundance, since based on CARD-FISH screening with probes for ANME-1 and Seep-SRB2, these taxa are highly abundant in the respective samples (see Fig. 1); 0 = < 0.5‰ of all archaeal and bacterial sequences.

Supplementary Figure 1. Development of sulfide production activity in the studied mesophilic G37 (A) and thermophilic G50 (B) AOM enrichment culture normalized to calculated dry weights. The slope of the regression line (calculated by least square fitting) was used to determine activity doubling times $T_d = \ln 2 / \ln(\Delta SP/\Delta t)$

Supplementary Figure 2. Relative sequence abundances of archaea (A) and bacteria (B) in ten long-term enrichment cultures under strict AOM conditions based on 16S rRNA tags of the variable regions V3-V5. Bubble sizes depict relative sequence abundances in %. AMV: Amon mud volcano, BSC: Black Sea seep, BSM: Black Sea microbial reef, MC: Menez Caldera seep, E20: Elba seep (20°C), GB37: Guaymas Basin seep (37°C), GB50: Guaymas Basin seep (50°C), GF: Gullfaks seep, GOM: Gulf of Mexico seep, HR: Hydrate Ridge seep. Next to ANME and their partner bacteria, AOM enrichment cultures cultivated on methane and sulfate for many years still contain a large number of other microorganisms with yet unknown function.
