# Supplementary figures and images for: Metabolic Capabilities of Microorganisms Involved in and Associated with the Anaerobic Oxidation of Methane
Source: Front Microbiol. 2016 Feb 2;7:46. doi: 10.3389/fmicb.2016.00046 (PMC4736303; doi:10.3389/fmicb.2016.00046)

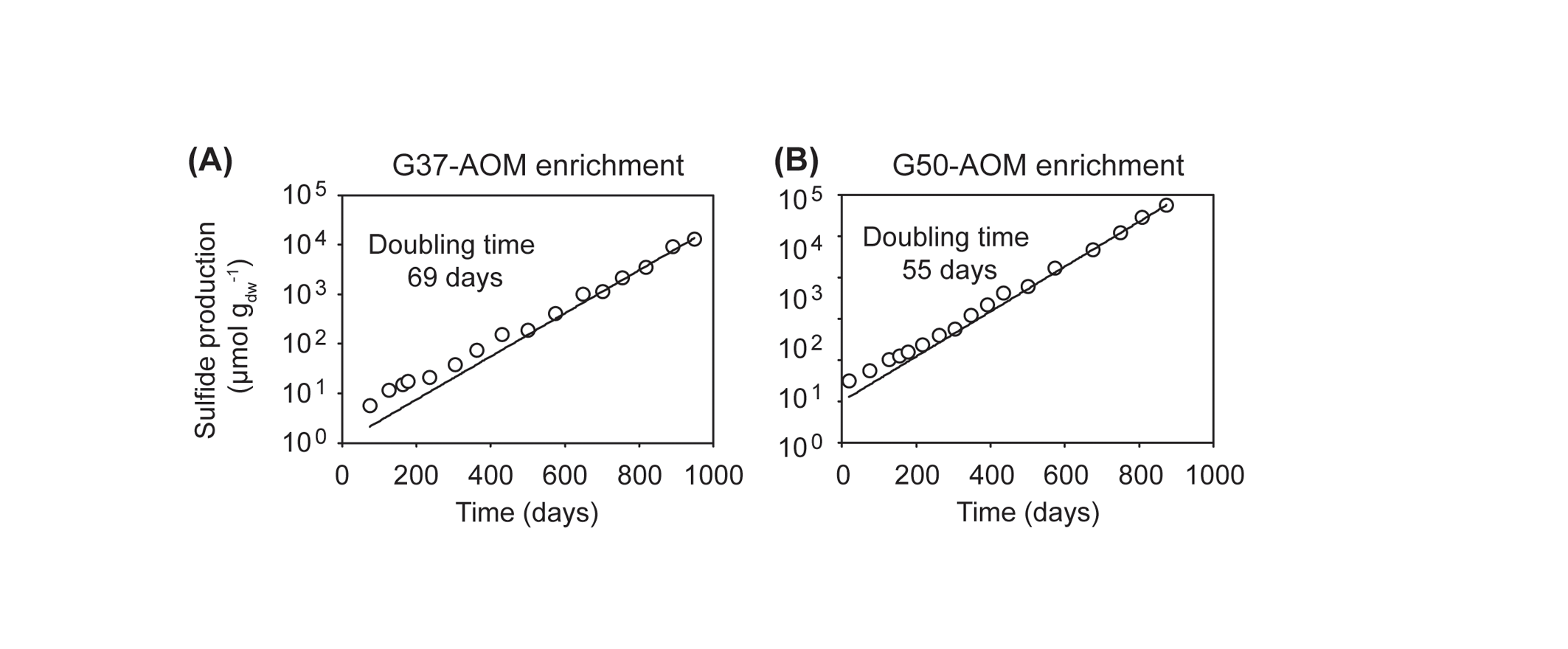

Supplement: Supplementary file 2 [file Image1.TIF]

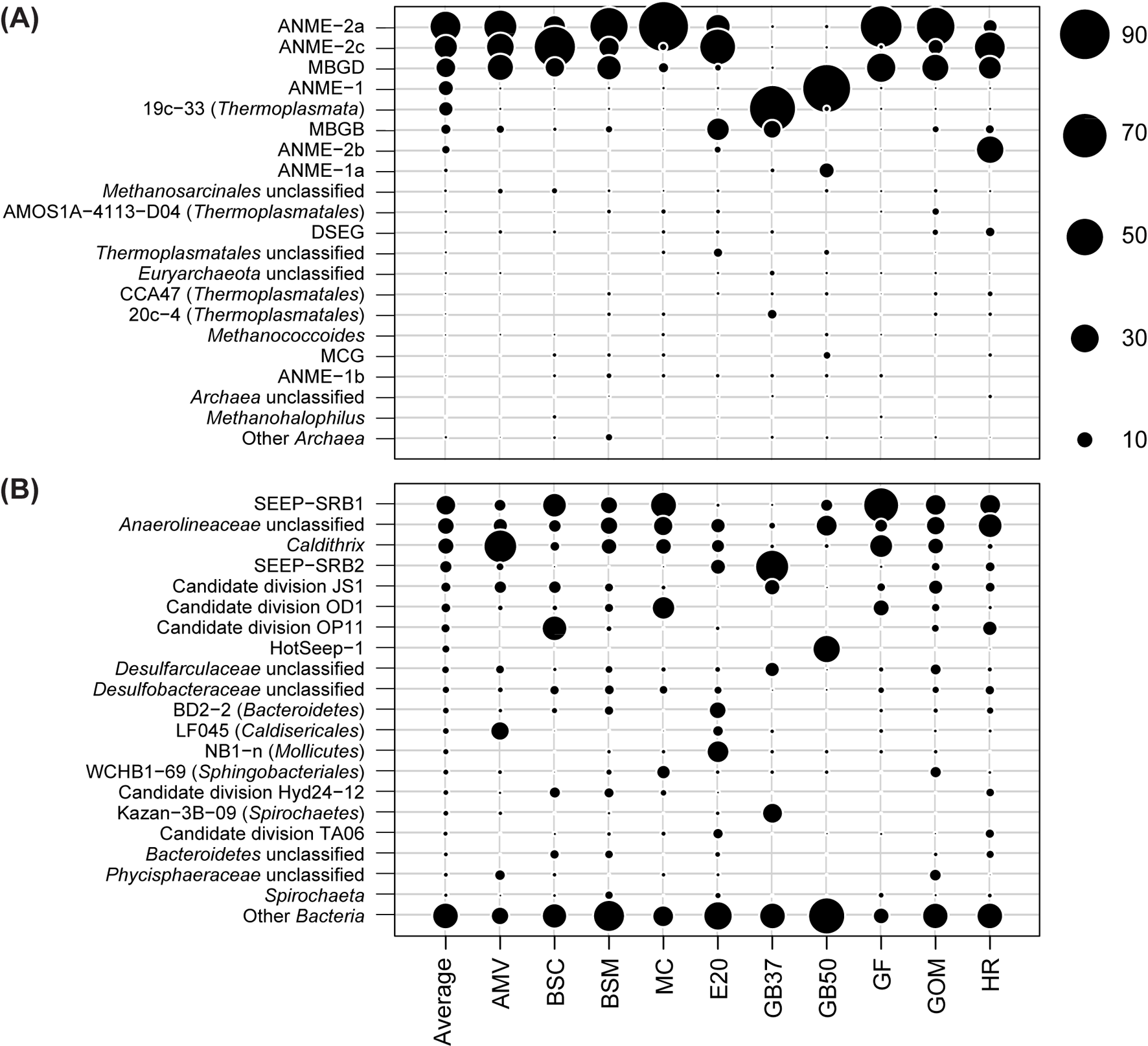

Supplement: Supplementary file 3 [file Image2.TIF]
